# The developmental transcriptome of the synanthropic fly *Chrysomya megacephala* and insights into olfactory proteins

**DOI:** 10.1186/s12864-014-1200-y

**Published:** 2015-01-23

**Authors:** Xiaoyun Wang, Mei Xiong, Chaoliang Lei, Fen Zhu

**Affiliations:** Hubei Insect Resources Utilization and Sustainable Pest Management Key Laboratory, Huazhong Agricultural University, Wuhan, 430070 China

**Keywords:** *Chrysomya megacephala*, Developmental transcriptome, Olfactory proteins, qPCR

## Abstract

**Background:**

*Chrysomya megacephala* (Fabricius) is a prevalent and synanthropic blowfly which has two sides, for being a pathogenic vector, an efficient pollinator, a promising resource of proteins, lipids, chitosan, biofuel et al., and an important forensic indicator. Moreover olfactory proteins are crucial component to function in related processes. However, the genomic platform of *C. megacephala* remains relatively unavailable. Developmental transcriptomes of eggs, larvae from 1st instar to before pupa stage and adults from emergence to egg laying period were built by RNA-sequencing to establish sequence background of *C. megacephala* with special lights on olfactory proteins.

**Results:**

Clean reads in eggs, larvae and adults were annotated into 59486 transcripts. Transcripts were assembled into 22286, 17180, 18934 and 35900 unigenes in eggs, larvae, adults and the combined datasets, respectively. Unigenes were annotated using Nr (NCBI non-redundant protein sequences), Nt (NCBI non-redundant nucleotide sequences), GO (Gene Ontology), PFAM (Protein family), KOG/COG (Clusters of Orthologous Groups of proteins), Swiss-Prot (A manually annotated and reviewed protein sequence database), and KO (KEGG Orthology). Totally 12196 unigenes were annotated into 51 sub-categories belonging to three main GO categories; 8462 unigenes were classified functionally into 26 categories to KOG classifications; 5160 unigenes were functionally classified into 5 KEGG categories. Moreover, according to RSEM, the number of differentially expressed genes between larvae and eggs, adults and eggs, adults and larvae, and the common differentially expressed genes were 2637, 1804, 2628 and 258, respectively. Among them, 17 odorant-binding proteins (OBPs), 7 chemosensory proteins (CSPs) and 8 ionotropic receptors (IRs) were differently expressed in adults and larvae. Ten were confirmed as significant differentially expressed genes. Furthermore, OBP *Cmeg32081-c4* was highly expressed in the female head and *Cmeg33593_c0* were up-regulated with the increase of larval age.

**Conclusions:**

A comprehensive sequence resource with desirable quality was built by comparative transcriptome of eggs, larvae and adults, enriching the genomic platform of *C. megacephala*. The identified differentially expressed genes would facilitate the understanding of metamorphosis, development and the fitness to environmental change of *C. megacephala*. OBP *Cmeg32081-c4* and *Cmeg33593_c0* might play a crucial role in the interactions between olfactory system and biological processes.

**Electronic supplementary material:**

The online version of this article (doi:10.1186/s12864-014-1200-y) contains supplementary material, which is available to authorized users.

## Background

*Chrysomya megacephala* is distributed worldwide across all the continents expect Antarctica. It is always found in association with humans or the activities of humans [1-4]. It is a common species found at fresh-food markets, garbage piles, restaurants, school cafeterias, hog and poultry farms and ranches [5]. Excessive populations are not only an irritant to humans but they can also be as disease vectors [6]. By crawling over and feeding on filth, flies become contaminated with pathogens that become entrapped on their legs and body surfaces or taken into the digestive tract with food. In subsequent visits to human food, the flies may leave behind some of these pathogens. Their habit of regurgitating some of their food and expelling feces frequently, both of which may contain pathogens, contaminate food, food preparation surfaces and storage containers [7].

In addition to causing annoyance and disease, *C. megacephala* is considered one of the most important species of flies to forensic science. It has long been used as forensic evidences to estimate postmortem interval (PMI) based on the morphological characteristics of individuals collected from the decomposing remains [8]. The ages of its larvae are commonly used as a keystone to achieve accurate PMI of bodies [9]. Therefore, extensive studies have been launched on the population dynamics, oviposition preference and development related studies of *C. megacephala* [10-15]. In modern facility agriculture *C. megacephala* is an important pollinator for orchards and vegetables, especially for mangos [16]. It is a top pollinator of Diptera which takes up 25% of all observed Orders in Northern Australia [17]. And in Guangxi province of China, *C. megacephala* accounts for over 30% of the total pollinators [18]. Besides, the larvae of *C. megacephala* are becoming a new sustainable resource for providing animal proteins, lipids, chitosan, and biofuel [19-22]. Despite all this, a key bottle neck to progress in controlling or using *C. megacephala* is lack of knowledge of the basic molecular biology of this species. Molecular progress of this species will provide important inroads to the discovery of novel target sites for population control, understanding of the immune response in this necrophagous fly. Transcriptome information and the differentially expressed genes related to lipometabolism in response to different kinds of oils were reported in larvae [23]. Despite efforts by developmental biologists, there is little molecular data regarding eggs and adults.

The olfactory system is usually used by insects to locate hosts, oviposition sites, and food sources. Completion of *Drosophila* genomes and progress in the study of *Drosophila* olfaction provided unprecedented opportunities to study other Dipterans olfaction. It has been clearly demonstrated that olfactory proteins, including the odorant-binding proteins (OBPs), chemosensory proteins (CSPs), odorant-degrading enzymes (ODEs), odorant receptors (ORs), ionotropic receptors (IRs), and sensory neuron membrane proteins (SNMPs), are involved in the peripheral events of odorant reception [24]. These olfactory proteins are critical for insects to move around and avoid risk factors; and to locate and evaluate food, shelter, mates, and breeding substrates [25]. For *C. megacephala*, olfactory proteins are vital for it to land premierly and colonize on corpses, and to locate the flowers precisely and feed on nectar, which provides a desirable pollination rate [17,26,27]. Identifying functional olfactory molecules will also facilitate development of attractants for baits in management systems.

In the present study, we used RNA-seq to dig the developmental stage-specific genes by building transcriptomes of eggs, larvae from 1st instar to before pupa stage, and adults from emergence to egg laying period (10 days old). We identified differentially expressed genes among eggs, larvae, and adults by comparative transcriptome analysis. We also screened olfactory proteins in this synanthropic fly, including OBPs, CSPs, and IRs, since the olfactory system is usually crucial for insects to locate hosts, oviposition sites, and food sources. Moreover, differential expressed OBPs and CSPs in larvae and adult were testified for transcriptome data validation.

## Results

### Illumina sequencing and assembly

Raw reads with low quality, adapter, and content of N more than 10% were excluded to achieve clean reads. Clean reads in eggs, larvae and adults of *C. megacephala* were 34716158, 34347518, and 35560603, respectively. All clean reads were assembled into transcripts by Trinity software; and the longest copy of redundant transcripts was regarded as a unigene [28,29]. Totally, 59486 transcripts were achieved and assembled into 35900 unigenes. Many unigenes had a length between 200–1000 bp (Table 1). Approximately 26.5% unigenes had a length more than 1000 bp and 12.5 % unigenes had a length more than 2000 bp (Table 1).

**Table 1:** **Number and length of transcripts and unigenes**

	**Transcripts**	**Unigenes**
200-500 bp	25787	20069
500-1 k bp	10636	6300
1 k-2 k bp	10038	5056
>2 k bp	13025	4475
Total number	59486	35900

### Annotation of unigenes

In order to annotate the unigenes, database Nr (NCBI non-redundant protein sequences), Nt (NCBI non-redundant nucleotide sequences), GO (Gene Ontology), PFAM (Protein family), KOG/COG (Clusters of Orthologous Groups of proteins), Swiss-Prot (A manually annotated and reviewed protein sequence database), and KO (KEGG Orthology) were used. Unigenes annotated in CE, CL, CA, CE-specific, CL-specific, CA-specific, Common and CE-CL-CA Combined datasets were 22286, 17180, 18934, 5505, 1711, 2721, 12809 and 35900, respectively (Table 2). For these datasets, number of unigenes annotated in different database and their separate percentage were counted. In the CE-CL-CA Combined dataset, the NR database (13797, 38.43%) had the largest match (Table 2). The SwissProt (10097, 28.12%), PFAM (11401, 31.75%), and GO (12196, 33.97%) shared similar quantities (Table 2).

**Table 2:** **Unigenes annotated in different databases**

	**CE**		**CL**		**CA**		**CE-specific**		**CL-specific**		**CA-specific**		**Common**		**CE-CL-CA combined**	
	**NO.**	**PCT (%)**	**NO.**	**PCT (%)**	**NO.**	**PCT (%)**	**NO.**	**PCT (%)**	**NO.**	**PCT (%)**	**NO.**	**PCT (%)**	**NO.**	**PCT (%)**	**NO.**	**PCT (%)**
NR	11155	50.05	10610	61.76	11123	58.75	770	13.99	485	28.35	862	31.68	9123	71.22	13797	38.43
NT	2774	12.45	2589	15.07	2693	14.22	111	2.02	52	3.04	63	2.32	2432	18.99	3035	8.45
KO	2956	13.26	2877	16.75	3003	15.86	116	2.11	79	4.62	142	5.22	2625	20.49	3418	9.52
Swissprot	8682	38.96	8441	49.13	8695	45.92	422	7.67	292	17.07	452	16.61	7044	54.99	10097	28.12
PFAM	9231	41.42	8732	50.83	9141	48.28	685	12.44	376	21.98	660	24.26	7565	59.06	11401	31.75
GO	9854	44.22	9281	54.02	9754	51.52	766	13.91	422	24.66	616	22.64	5013	39.14	12196	33.97
KOG	6759	30.33	6542	38.08	6789	35.86	242	4.40	154	9.00	288	10.58	6007	46.90	7588	21.13
Total NO.	22286		17180		18934		5505		1711		2721		12809		35900	

*Abbreviations:* CE: Unigenes of *Chrysomya megacephala* eggs; CL: Unigenes of *C. megacephala* larvae; CA: Unigenes of *C. megacephala* adults; CE-specific: Specific unigenes of *C. megacephala* eggs; CL-specific: Specific unigenes of *C. megacephala* larvae; CA-specific: Specific unigenes of *C. megacephala* adults; Common: Common unigenes of *C. megacephala* eggs, larvae and adults; CE-CL-CA Combined: Total unigenes of *C. megacephala* eggs, larvae and adults.

NO.: number; PCT (%): percentage (%); NR: NCBI non-redundant protein sequences; NT: NCBI non-redundant nucleotide sequences; KO: KEGG Orthology; Swissprot: A manually annotated and reviewed protein sequence database; PFAM: Protein family; GO: Gene Ontology; KOG: Clusters of Orthologous Groups of proteins; Total NO.: Total number of annotated unigenes.

**Figure 1:**
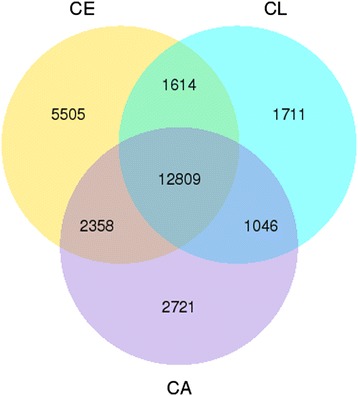
**Venn diagram of the number of unigenes with reads per kilo bases per million mapped (RPKM) > 0.3 in CE, CL, and CA.** CE: *Chrysomya megacephala* eggs, CL: *C. megacephala* larvae, CA: *C. megacephala* adults.

Unigenes annotated in eggs, larvae, and adults were shown in supplementary materials with information of gene’s ID, length, reads per kilo bases per million mapped (RPKM) and annotation to different databases (Additional files 1, 2 and 3). The number of the unigenes with RPKM > 0.3 shared by eggs and larvae, larvae and adults, and eggs and adults were 14423, 13823, and 15167, respectively. Eggs, larvae, and adults had 12809 common unigenes (Figure 1).

### Functional annotation results

**Figure 2:**
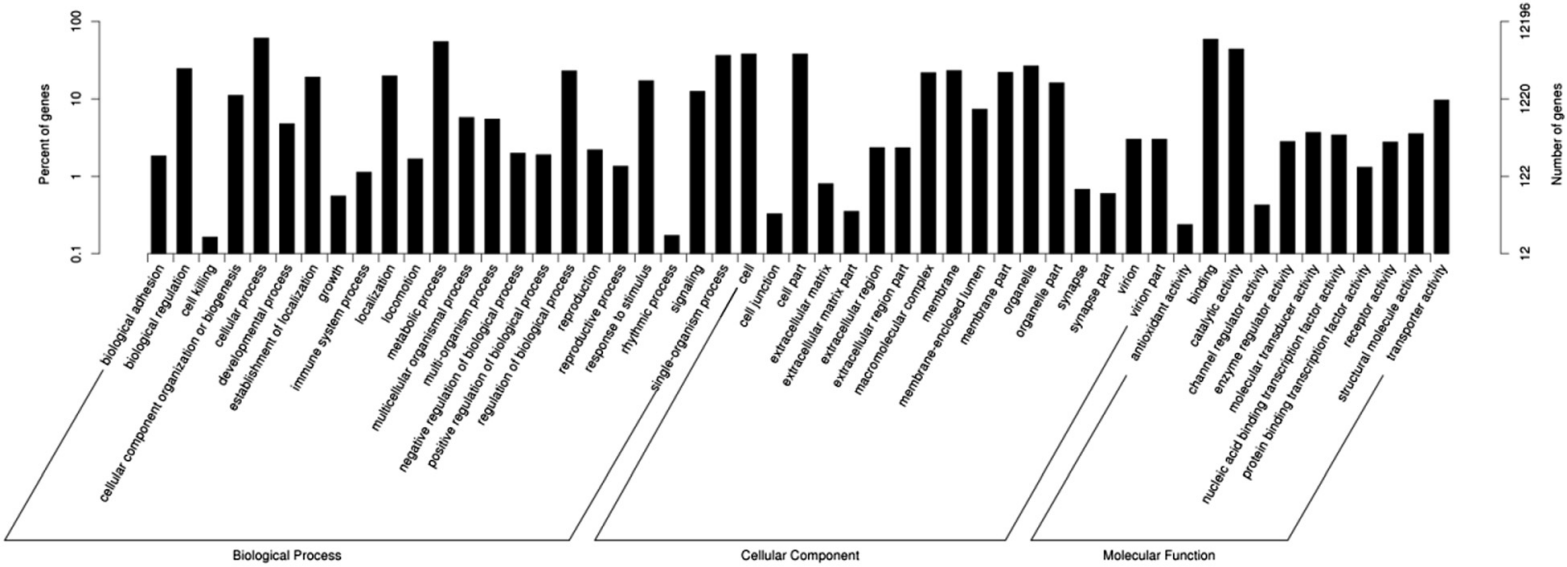
**Histogram of GO classifications of unigenes.**

Totally 12196 were annotated into 51 sub-categories belonging to three main GO categories: biological process (BP), cellular component (CC), and molecular function (MF) (Figure 2). There were 23 sub-categories in BP, 17 sub-categories in CC, and 11 sub-categories in MF. Top ten sub-categories were cellular process (7398), metabolic process (6668), single-organism process (4423), biological regulation (2986), cell (4612), cell part (4611), organelle (3235), membrane (2815), binding (7161), and catalytic activity (5343) (Additional file 4).

**Figure 3:**
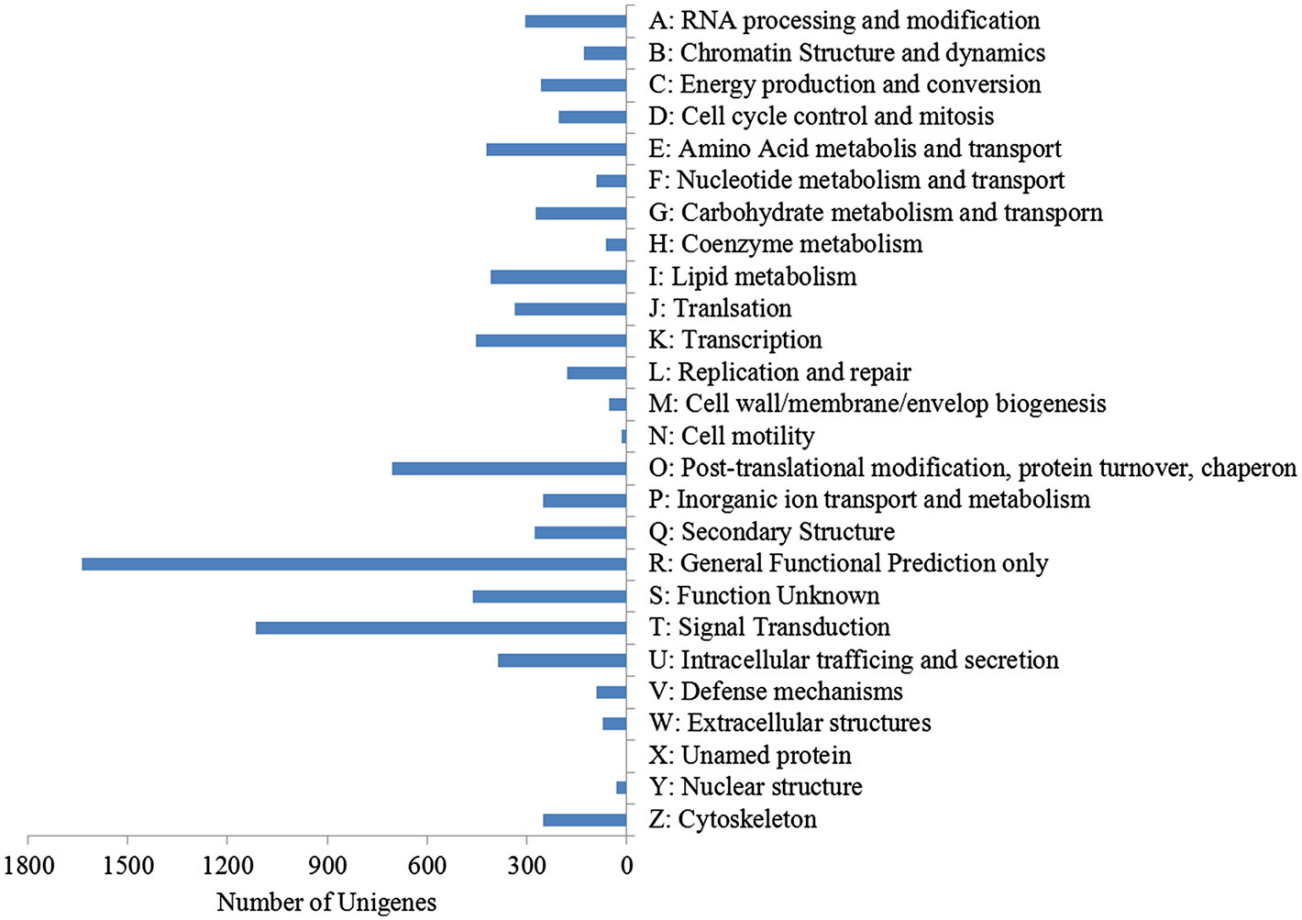
**Histogram of KOG classifications of unigenes.**

By KOG classifications, 8462 unigenes were classified functionally into 26 categories (Figure 3). The cluster of ‘General Functional Prediction only’ was the largest group, which had 1639 unigenes. The group of ‘Signal transduction’ was in second place, which had 1115 unigenes. Top 2 categories had 32.5% of unigenes annotated to KOG database (Additional file 5).

**Figure 4:**
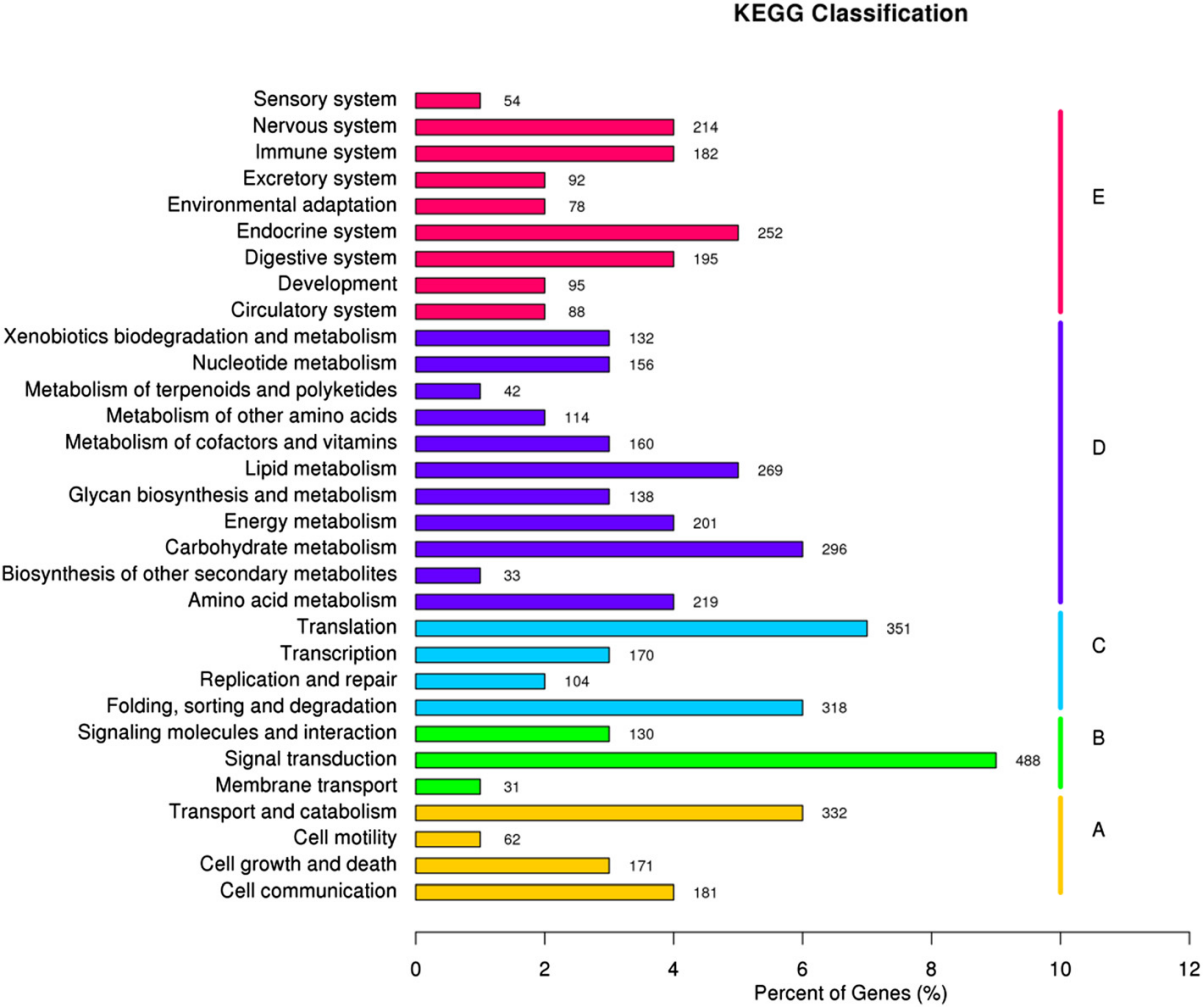
**Histogram of KEGG classifications of unigenes.** A: Cellular Processes, B: Environmental Information Processing, C: Genetic Information Processing, D: Metabolism, E: Organismal Systems.

In total, 5160 unigenes were functionally classified into 5 KEGG categories (Figure 4). They were cellular processes (746 unigenes, 13.95% of the unigenes annotated to the KEGG database), environmental information processing (649, 12.14%), genetic information processing (943, 17.63%), metabolism (1760, 32.91%), and organismal systems (1250, 23.37%) (Additional file 6). Among 31 sub-category, ‘signal transduction’, ‘translation’, ‘transport and catabolism’ were the top 3.

### Differentially expressed genes

**Figure 5:**
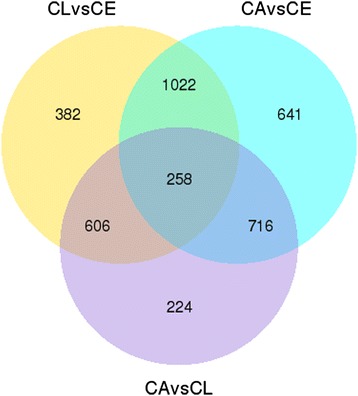
**Venn diagram of the number of differentially expressed genes in CE, CL, and CA.** Differentially expressed genes were selected by log_2_ Fold change > 1 and q value < 0.005 according the method of Storey et al. [30]. CE: *Chrysomya megacephala* eggs, CL: *C. megacephala* larvae, CA: *C. megacephala* adults.

**Figure 6:**
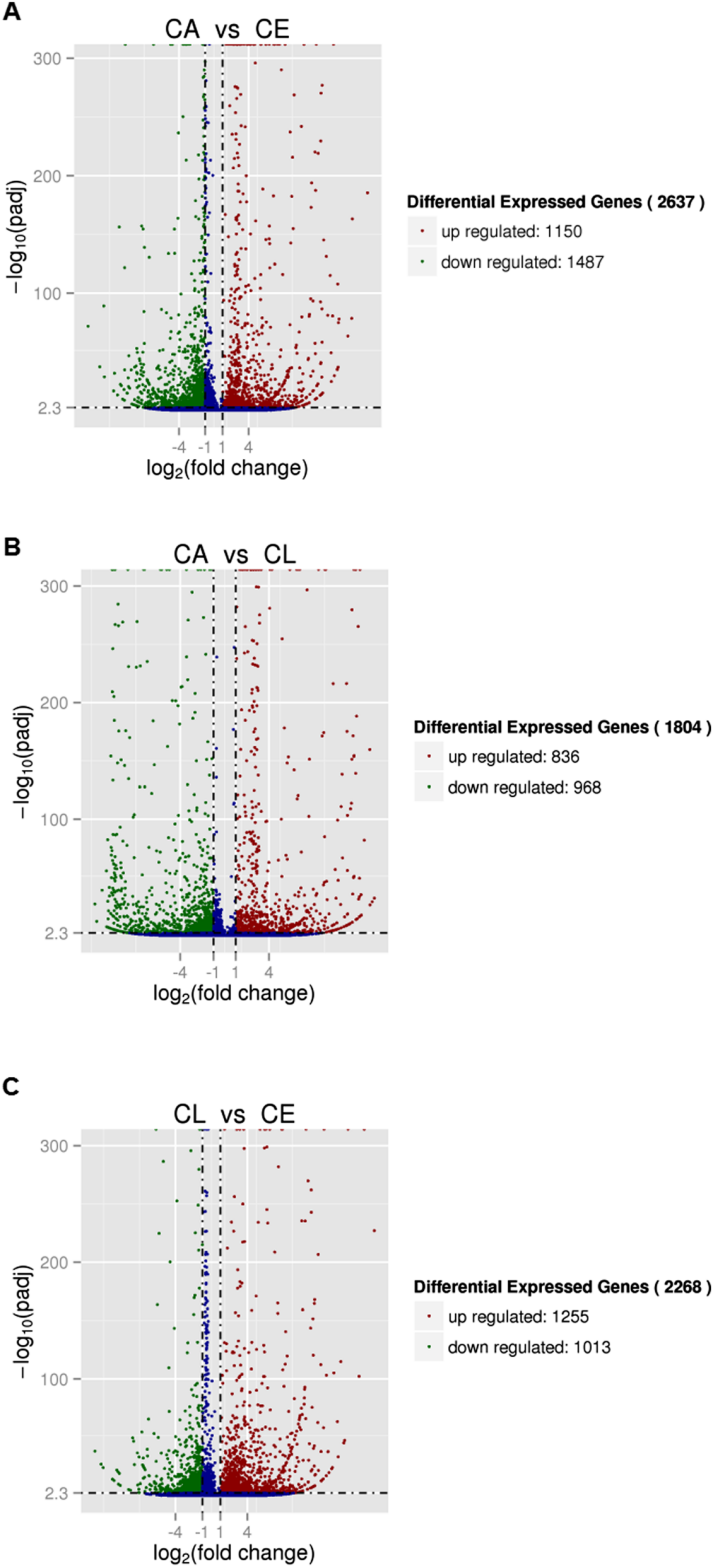
**Volcano plot of differentially expressed genes in eggs, larvae, and adults. A**: Volcano plot of differentially expressed genes between CA and CE. **B**: Volcano plot of differentially expressed genes between CA and CL. **C**: Volcano plot of differentially expressed genes between CL and CE. Differentially expressed genes were selected by log2 Fold change > 1 and q value < 0.005 according the method of Storey et al. [30]. Splashes represent different genes. Blue splashes means genes without significant different expression. Red splashes means significantly up expressed genes. Green splashes means significantly down expressed genes. CE, CL, and CA represent eggs, larvae and adults of *Chrysomya megacephala,* respectively*.*

Differentially expressed genes were selected by RSEM with conditions of log_2_ Fold change > 1 and q value < 0.005 [30]. The number of differentially expressed genes between larvae and eggs, adults and eggs, and adults and larvae were 2637, and 1804, 2628, respectively (Additional files 7, 8 and 9). There were 258 common differentially expressed genes among eggs, larvae, and adults (Figure 5). Totally, 1280 differentially expressed genes in larvae and eggs were also differential expressed in adults and eggs. And 864 differentially expressed genes in larvae and eggs were also differential expressed in adults and larvae (Figure 5). We also found that 974 differentially expressed genes in adult and egg were differential expressed in adults and larvae (Figure 5). More expressed genes in larvae than in eggs, in adults than in eggs, and in adults than in larvae were 1255, 1150, and 836, respectively (Figure 6). But less expressed genes in larvae than in eggs, in adults than in eggs, and in adults than in larvae were 1013, 1847, and 968, respectively (Figure 6).

### Expression profiles of olfactory proteins

We identified 49 OBPs, 12 CSPs, and 11 IRs through Nr database (Nucleotide sequences were listed in the Additional files 10, 11 and 12). Seventeen OBPs, 7 CSPs, and 8 IRs were differently expressed in adults and larvae (Table 3). Only 9 OBPs and 1 CSP were significantly differently expressed (Table 3).

**Table 3:** **Differential expressed olfactory-related genes in adults and larvae**

	**Gene**	**Readcount_Adult**	**Readcount_Larva**	**log**_**2**_ **Fold_change**	**q**
OBPs	Cmeg21243_c0	2.286	1.4019	0.7054	>0.005
	Cmeg21269_c0	32.7183	854.4521	−4.7068	<0.005*
	Cmeg21549_c0	5.2439	0.163	5.0076	>0.005
	Cmeg21654_c0	0.9608	2.3474	−1.2888	>0.005
	Cmeg23484_c0	219.3585	95.1018	1.2057	<0.005*
	Cmeg24919_c0	71.1444	49.996	0.5089	>0.005
	Cmeg25217_c1	1.1264	0.0326	5.1106	>0.005
	Cmeg27557_c0	4.0088	4.0754	−0.0238	>0.005
	Cmeg28108_c1	0.762	12.9442	−4.0864	<0.005*
	Cmeg28677_c0	14.3274	2.6083	2.4576	>0.005
	Cmeg29057_c0	11.9602	107.3274	−3.1657	<0.005*
	Cmeg30479_c0	5.8973	231.3797	−5.2941	<0.005*
	Cmeg31019_c2	9.163	94.0392	−3.3594	<0.005*
	Cmeg32081_c4	20.6073	0.4891	5.397	<0.005*
	Cmeg33593_c0	788.7731	2558.267	−1.6975	<0.005*
	Cmeg8311_c0	303.3185	477.3415	−0.6542	>0.005
	Cmeg8717_c0	2.1535	50.6347	−4.5554	<0.005*
CSPs	Cmeg21206_c0	1559.024	361.927	2.1069	<0.005*
	Cmeg25565_c0	2.1402	0.0529	5.3383	>0.005
	Cmeg23554_c0	29.416	33.3811	−0.1824	>0.005
	Cmeg349412_c0	0.8719	0.0265	5.0428	>0.005
	Cmeg30884_c0	3.8048	4.0769	−0.0997	>0.005
	Cmeg5343_c0	0.0793	0.1058	−0.4166	>0.005
	Cmeg645582_c0	0.1585	0.0265	2.5834	>0.005
	Cmeg386817_c0	0.1982	0.3174	−0.6797	>0.005
	Cmeg425837_c0	0.1982	0.2116	−0.0947	>0.005
	Cmeg23554_c0	29.416	33.3811	−0.1824	>0.005
IRs	Cmeg1881_c0	0.1585	0.5026	−1.6645	>0.005
	Cmeg20304_c0	0.1982	0.0529	1.9053	>0.005
	Cmeg22717_c0	0.1189	0.0265	2.1684	>0.005
	Cmeg25409_c2	0.1585	0.3703	−1.224	>0.005
	Cmeg25409_c3	0.1982	0.3968	−1.0016	>0.005
	Cmeg18139_c0	0.0793	0.2381	−1.5865	>0.005
	Cmeg3349_c1	0.0793	0.1587	−1.0016	>0.005
	Cmeg475644_c0	0.4756	0.3174	0.5834	>0.005

Note: q value was calculated according the method of Storey et al., 2003. *q < 0.005 meant significantly different.

### Validation of transcriptome data by qPCR

**Figure 7:**
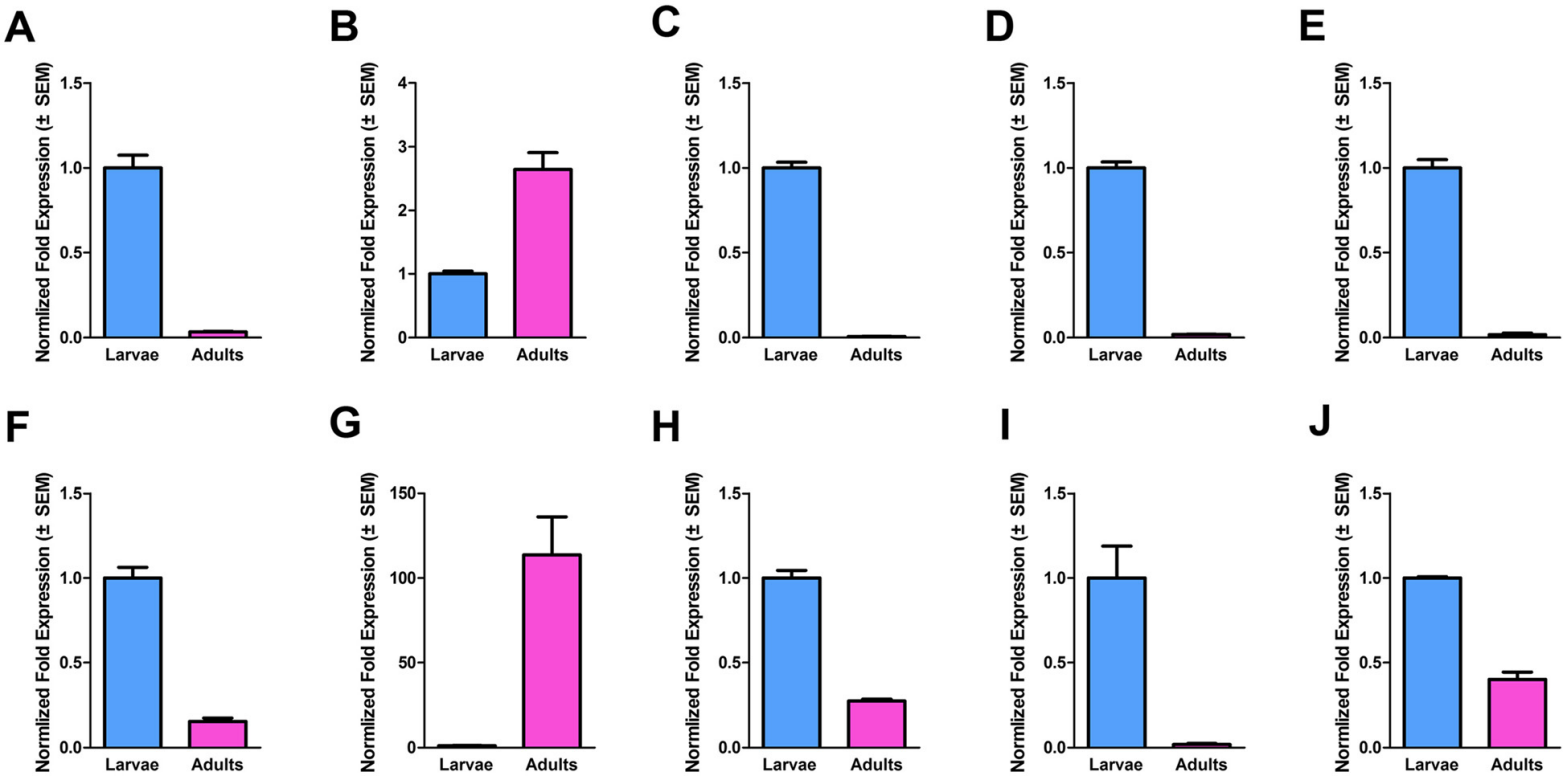
**qPCR results of differentially expressed genes in larvae and adults.** The expression levels of the mix-aged larva and mix-aged adult were showed by blue and red purple bar, respectively by the results of 2^-ΔΔCT^ method with three biological repeats. Sub-caption A to J indicate the identified different expressed genes between the larvae and adults (**A**: *Cmeg21269_c0* **B**: *Cmeg23484_c0* **C**: *Cmeg28108_c1* **D**: *Cmeg29057_c0* **E**: *Cmeg30479_c0* **F**: *Cmeg31019_c2* **G**: *Cmeg32081_c4* **H**: *Cmeg33593_c0* **I**: *Cmeg8717_c0* **J**: *Cmeg21206_c0*). Significant difference was detected in all the 10 genes (p < 0.01).

**Figure 8:**
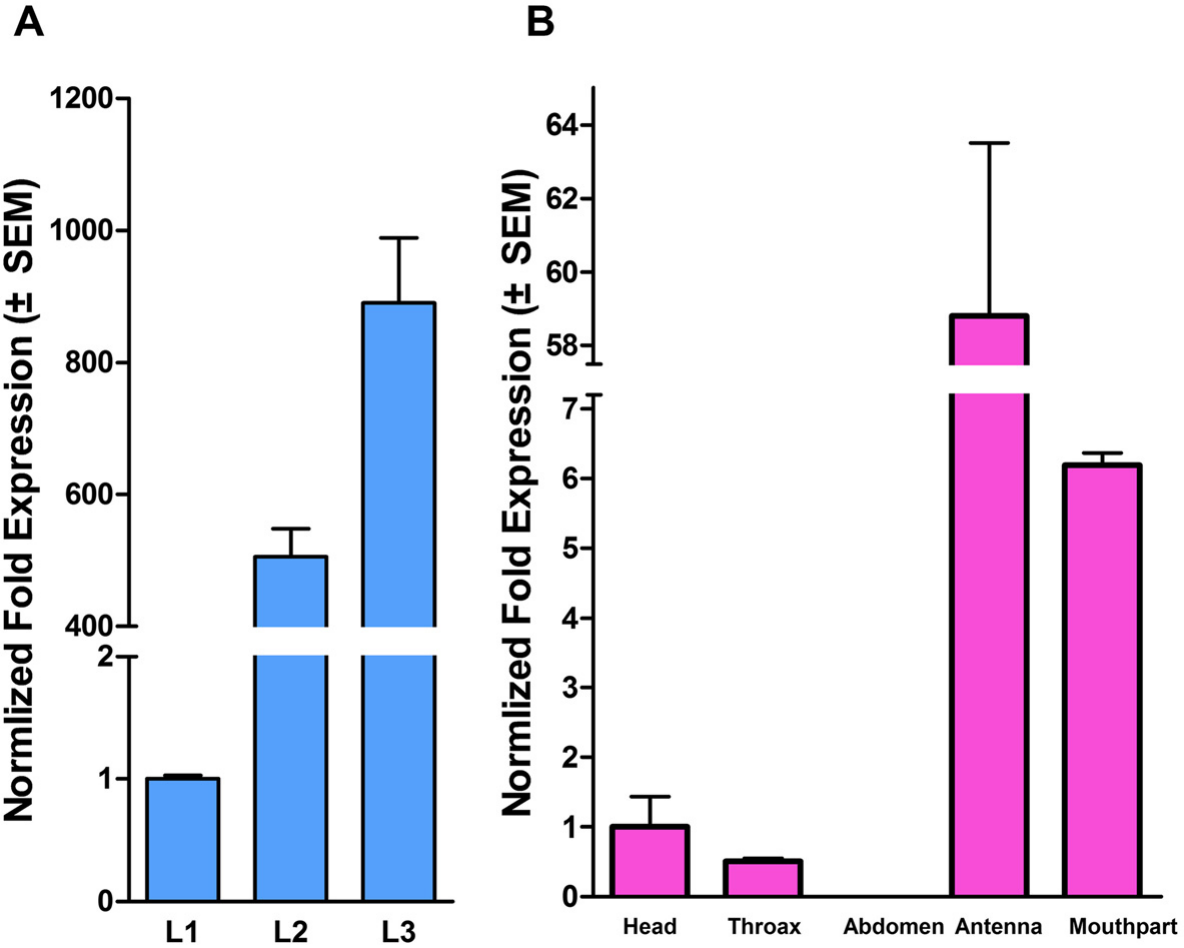
**Developmental expression patterns of** ***Cmeg33593_c0*** **in** ***C. megacephala*** **larvae and tissue-specific expression patterns of** ***Cmeg32081_c4*** **in** ***C. megacephala*** **females.** The expression levels of *Cmeg33593_c0* in different instar of larvae were showed red purple bar by the results of 2^-ΔΔCT^ method with three biological repeats **(A)**. The expression levels of *Cmeg32081_c4* in various tissues of female were showed red purple bar by the results of 2^-ΔΔCT^ method with three biological repeats **(B)**. And the column title L1, L2 and L3 represent 1st, 2nd and 3rd instar of larvae, respectively.

To validate the transcriptome result, we selected 10 significant differentially expressed genes from Table 2 for quantitative real-time PCR (qPCR) conformation. The primers used for qPCR were shown in Additional file 13. The result of qPCR was shown in Figure 7. Seven OBPs transcripts and one CSP transcript which have demonstrated by RNA-seq to be enriched in larvae were confirmed by qPCR. The expression proportion of the 8 genes in adult to larva varied from the least 1.6% (OBP *Cmeg31019_c2,* Figure 7F) to the biggest 40.15% (CSP *Cmeg21206_c0*, Figure 7J). Additionally, RNA-seq data for two OBPs, *Cmeg23484-c0* and *Cmeg32081-c4*, enriched in adults mirrored the qPCR data (Figure 7). *Cmeg23484-c0* and *Cmeg32081-c4* had significantly higher transcriptional level in adult than in larva with 2.64 and 113.67 fold exchanges, respectively. Moreover, the tissue-specific expression pattern of *Cmeg32081-c4* in female and the larval developmental expression pattern of *Cmeg33593-c0* were performed (Figure 8). OBP *Cmeg32081-c4* was highly expressed in the head of the female (Figure 8B) and *Cmeg33593_c0* were up-regulated with the increase of larval age (Figure 8A).

## Discussion

### Overview of transcriptome data

Developmental transcriptomes were established of eggs, mix-aged larvae, and mix-aged adults, providing a relatively comprehensive gene pool of *C. megacephala.* The number of clean reads in larva transcriptome was 34347518, which was similar to that of a reported larval transcriptome of *C. megacephala* [31]. And the number of clean reads from egg and adult transcriptome were 34,716,158, and 35,560,603, respectively. All these clean reads were assembled into 59486 transcripts by Trinity software. Transcripts were assembled into 22286, 17180, 18934 and 35900 unigenes in eggs, larvae, adults and the combined datasets, respectively. A total of 35900 unigenes were annotated by Nr, Nt, GO, PFAM, KOG/COG, Swiss-Prot, and KO. Moreover, thousands of different expressed and common genes between larvae and eggs, adults and eggs, adults and larvae and all three stages were harvested, which both facilitate future developmental and evolutionary studies of *C. megacephala*, and contribute to future work in blowfly comparative genomic. Ten of the identified differentially expressed genes were validated by qPCR, showing that the quality of the transcriptome was desirable.

### Olfactory proteins

Striking similarities span a phylogenetically broad array in olfaction of insects, implying that there is an optimal solution to the problem of detecting and discriminating odors [32]. Therefore, the research into the parallel OBPs in *Drosophila melanogaster* would provide valuable information to the link the biological roles into the candidate OBPs [25]. Moreover, olfactory proteins have been illustrated to act in the insect nutrient uptake, life span and behavior change during developmental stages [33,34]. The developmental transcriptome of *C. megacephala* would be an opportunity to understand the interactions between olfactory proteins anddevelopment. Totally 49 OBPs, 12 CSPs, and 11 IRs were identified. Moreover, a phylogenetic wheel was made based on (deduced) amino acids from *D. melanogaster* OBPs and *C. megacephala* OBPs (Additional files 14 and 15), since the identified OBPs had the largest number. We also identified some ODEs, ORs and SNMPs, however they were relatively incomplete, therefore we did not take them out for further analysis. This might be rooted into the sampling characteristics and the abundance of the related genes. Seventeen OBPs, 7 CSPs, and 8 IRs were differently expressed in adults and larvae, since the olfactory systems function mostly in adults and larvae.

Among the 10 significant different olfactory genes, two OBPs genes were more abundant in adults than in larvae. One is *Cmeg23484-c0*, and the other is *Cmeg32081-c4. Cmeg23484-c0* showed 91% identities with *Calliphora stygia* OBP (AID61300), 91% identities with *Delia antiqua* OBP (BAN59723), and 64% identities with *D. melanogaster* OBP44a DmelOBP19d (Alignment results were showed in the Additional file 16). BAN59723 were functionally annotated as an insect pheromone/odor binding protein domains. And *Dmel*OBP44a of adult decreased in expression with increasing organism age, which was considered to be a link between the olfactory sensation and aging [35]. *Dmel*OBP44a was detected in the female antenna extract but not male, which might reflect true sexual dimorphism in the expression of OBPs [36]. The other one *Cmeg32081-c4*, which showed highest fold changes, was highly expressed in female head, especially in antenna and mouthpart (Figure 8B). *Cmeg32081-c4* showed 71% identities with *C. stygia* OBP (AID61308) and 38% identities with of *Dmel*OBP19d (ACY93747) (Alignment results were showed in the Additional file 16). Both *Dmel*OBP44a and *Dmel*OBP19d are the most abundant OBPs in adult antenna extracts by LC/MS/MS [36,37]. *Dmel*OBP19d was also expressed in the head at different levels and was considered to have a close connection to the variation in life span associated with nutrient sensing and synaptic transmission by network analysis [33,38]. The function of *Dmel*OBP44a and *Dmel*OBP19d should have an instructive role for the research of *Cmeg23484-c0* and *Cmeg32081-c4* in aging and nutrient sensing.

Seven OBPs and 1 CSP were found more abundant in larvae than in adults. Among them, OBP *Cmeg33593_c0* has the highest RPKM value, indicating that it has the highest expression level in larvae [39]. *Cmeg33593_c0* showed 88% identities with OBP *C. stygia* AID61305 and 56% identities with *Dmel*OBP99b (ABW78474) (Alignment results were showed in the Additional file 16). However, the expression of *Dmel*OBP99b has been well documented in various developmental and physical situations in adults. *Dmel*OBP99b was found to be more abundant in females than males [40]. And *Dmel*OBP99b showed strong adult-biased expression and altered expression levels during aging in both sexes, but in opposite directions: the expression level of young virgin *Drosophila* females was lower than that of the old ones, while the expression level of the young virgin males was higher than that of the old ones [41]. For males, *Dmel*OBP99b was up-regulated after courting females and down and regulated after mating [41,42]. Moreover, *Dmel*OBP99b was down-regulated after being starved [43]. Therefore, *Dmel*OBP99b was suggested to be sensitive to and probably influence nutrient status and reproductive status in both males and females [44]. For example, the ectopic expression of *Dmel*OBP99b could reduce virgin female receptivity and copulation frequency [45]. Moreover, *Dmel*OBP99b in adults has a wide and comprehensive influence in aversive tastants uptake, which should have evolved to prevent ingestion of toxic compounds [46]. In the *UAS-OBP99b-RNAi* adults, the consumption of berberine and papaverine compared to the control were raised higher by 47% respectively, and the coumarin and denatonium were decresed by 23% and 40% respectively [46]. In addition, the combinatorial response profiles in females and males were diverse for intake of bitter tastants with this line [46]. The discriminative binding profiles between sexes should have a natural tie with the varied expression patterns of *Dmel*OBP99b, which calls for more experiments, guiding the behavior of males and females especially during the copulation.

There should be a certain relationship between OBPs and ORs in odorant detection by comparing behavioral response profiles of OBPs and molecular response profiles of odorant receptors and features of functional organization emerge between behavioral response profiles of OBPs and electrophysiological response profiles of odorant receptors [37,47,48]. And the binding function of *Dmel*OBP99b has been well studied. In male it is responsible for the binding of E2-hexenal, acetophenone, benzaldehyde, citral and d-carvone, while in female it is responsible for 2-ethylpyrazine, acetophenone, benzaldehyde, citral and d-carvone [37]. For example, OR10a and OR67a are activated by acetophenone and benzaldehyde, which were identified as bioactive compounds of most floral volatiles [49]. And the behavior response is affected by the suppression of *Dmel*OBP99b for both females and males. And benzaldehyde and E2-hexenal were identified from the volatile organic compounds (VOCs) [50,51]. And acetophenone and benzaldehyde both have a similar structure feature of benzoyl chemical groups [37]. So probe into *Cmeg33593_c0* might help to understand the localization of hosts, oviposition sites and food sources, mating behavior and the connection between OBPs and life span.

Though *Dmel*OBP99b in adult was well studied, the expression and function of larvae were scarce. While, it is interesting that the expression of *Dmel*OBP99b-like OBP *Cmeg33593_c0* increased with larval growth, which was found occasionally (Figure 8A). It seems that *Cmeg33593_c0* was accumulated during larval stages and consumed in adults. According to our observations, the odors from the feeding container increased during the sampling days of larvae. It could be easily explicable because the feedstuff (fish meat) decayed gradually and deeply. Then how to understand the connections between the denser odor and the increasing *Cmeg33593_c0*? Would *Cmeg33593_c0* be a protective amino acid to eliminate the affect of the offensive VOCs, since the parallel of *Cmeg33593_c0*, *Dmel*OBP99b has a broad odor-binding profile? And anther explanation: together with all information of *Dmel*OBP99b in adult, the increase of *Cmeg33593_c0* during larval stages might be bound up with aging, the nutrient accumulation and feeding behavior, which might play a role in adult physiological status. More experiments are needed to illustrate the crosstalk between the olfactory proteins and the developmental genes. The probe into *Cmeg33593_c0* is to throw out a minnow to catch a whale. More significant work could be launched by the established sequence platform, which would facilitate the illustration of the crosstalk between the olfactory proteins and development, the application of pollination and forensic science in *C. megacephala*, avoid potential transmission of pathogens.

## Conclusions

A comprehensive sequence resource with desirable quality was built by developmental transcriptomes of eggs, larvae and adults, enriching the genomic platform, which would facilitate the understanding of metamorphosis, development and the fitness to environmental change of *C. megacephala*. The identified OBP *Cmeg32081-c4* and *Cmeg33593_c0* might play a crucial role in the interactions between olfactory system and physiological status.

## Methods

### RNA sequencing

Eggs, mixed larvae from 1st instar to before pupa stage, and mixed adults from emergence to egg laying period (10 days old) were prepared for RNA extraction. RNA purity was checked using the NanoPhotometer® spectrophotometer (IMPLEN, CA, USA). And RNA integrity was assessed using the RNA Nano6000 Assay Kit of the Agilent Bioanalyzer 2100 system (Agilent Technologies, CA, USA). A total amount of 3 μg RNA per sample was used as input material for the RNA sample preparations. Briefly, mRNA was purified from total RNA using poly-T oligo-attached magnetic beads. Sequencing libraries were generated using NEBNext® Ultra™ RNA Library Prep Kit for Illumina® (NEB, USA) following manufacturer’s recommendations and index codes were added to attribute sequences to each sample. Library quality was assessed on the Agilent Bioanalyzer 2100 system. The clustering of the index-coded samples was performed on a cBot Cluster Generation System using TruSeq PE Cluster Kit v3-cBot-HS (Illumia) according to the manufacturer’s instructions. After cluster generation, the library preparations were sequenced on an Illumina Hiseq2000 platform and 100 paired-end reads were generated.

### Transcriptome data analysis

Raw data (raw reads) of fastq format were firstly processed through in-house perl scripts. Clean reads were obtained after removing reads that contained adaptor sequences, reads in which more than 10% of the bases were unknown, and reads in which more than 50% of the quality values of the bases were less than 5. At the same time, Q20, Q30, GC-content and sequence duplication level of the clean data were calculated. All the downstream analyses were based on clean data with high quality. The left files (read1 files) from all libraries/samples were pooled into one big left.fq file, and right files (read2 files) into one big right.fq file. Transcriptome assembly was accomplished based on the left.fq and right.fq using Trinity with min_kmer_cov set to 2 by default and all other parameters set default [28,29]. Unigenes were used for BLAST searches with annotation against the Nr database using an E-value cut-off of 10^−5^ (E-value < 0.00001). After sequence assembly, the unigene sequences were also aligned by BLASTX to protein databases such as Swiss-Prot, KEGG and COG, in order to retrieve proteins with the highest sequence similarity to the given unigenes along with putative functional annotations. Gene function was annotated based on the following databases: Nr, Nt, Pfam, KOG/COG, Swiss-Prot, KO and GO.

### qPCR

Total RNA was extracted from 1st, 2nd and 3rd instar larvae for larval stage expression. Adult females on ice were quickly dissected into head (without antenna and mouthpart), thorax, abdomen, antenna and mouthpart. Tissues were immediately transferred into liquid nitrogen before RNA extraction. Total RNA from each sample was extracted using TRIzol® Reagent (Ambion®, Life technologies, U.S.) according to the manufacturer’s protocol. Concentration and quality of each RNA sample was determined by Nanodrop2000 (Thermo Scientific, U.S.). Samples were allowed into further experiments with an appropriate OD260/280 value from 1.9 to 2.1. One μg of total RNA from each sample was applied to produce the first strand cDNA with First Strand cDNA Synthesis Kit (NEWBIO Tech., Canada) according to the manufacturer’s protocol. All cDNA was stored at −20°C before use.

The primers were designed with IDT online tools (http://www.idtdna.com/scitools/Applications/RealTimePCR/) and listed in the Additional file 13. RT-PCR was applied to test the primers’ quality of differentially expressed genes. Primer pairs led to the PCR products exact identities between the RT-PCR fragments and transcripts from RNA-seq were used for qPCR. RT-PCR was performed as follows: 95°C for 3 min, 35 cycles at 95°C for 30 sec, 57°C for 30 sec and 72°C for 20 sec; and final extension at 72°C for 5 min. qPCR was performed using Real Master Mix (SYBR Green) (NEWBIO Tech., Canada) on a Bio-Rad iQ5 Optical System (Bio-Rad). The procedure for qPCR were as follows: initial denaturation temperature, 95°C for 30 sec, followed by 40 cycles at 95°C for 5 sec and 59°C for 30 sec, and 72°C for 2 min to terminate the reaction. After the reaction, a melting curve analysis from 55°C to 95°C was applied to ensure consistency and specificity of the amplified product. Gene *α-tubulin* (GenBank: KM289152) was used as positive control to test the quality of cDNA. *Elongation factor 1* (EMBL: FR719225) and *RPL8* (GenBank: KM289151) of *C. megacephala* were used as reference genes in qPCR of *Cmeg32081-c4* and *Cmeg33593_c0*, respectively (according to our previous work, unpublished). For each treatment, three biological replicates were conducted. Data of qPCR was analyzed by 2^-ΔΔCT^ method.

## Additional files

Additional file 1:
**S1-annotation Eggs.**
Additional file 2:
**S2-annotation Larvae.**
Additional file 3:
**S3-annotation Adults.**
Additional file 4:
**S4-SC1 GO.**
Additional file 5:
**S5-SC2 KOG.**
Additional file 6:
**S6-SC3 KEGG.**
Additional file 7:
**S7-SD1 CAvsCE.**
Additional file 8:
**S8-SD2 CAvsCL.**
Additional file 9:
**S9-SD3 CLvsCE.**
Additional file 10:
**S11-nucleotide sequences of OBPs.**
Additional file 11:
**S12-nucleotide sequences of CSPs.**
Additional file 12:
**S13- nucleotide sequences of IRs.**
Additional file 13:
**S10-Primers of qPCR for** ***C. megacephala*** **used in the experiment.**
Additional file 14:
**S14-deduced amino acid sequences of the identified OBPs.**
Additional file 15:
**S15-Phylogenetic analysis of OBPs of** ***Chrysomya megacephala*** **and** ***Drosophila melanogaster.***
Additional file 16:
**S16-Mutiple alignment of deduced amino acid of the** ***Cmeg23484_c0*** **,** ***Cmeg32081_c4*** **and** ***Cmeg33593_c0*** **.**
