# Supplementary material for: The developmental transcriptome of the synanthropic fly Chrysomya megacephala and insights into olfactory proteins
Source: BMC Genomics. 2015 Jan 23;16(1):20. doi: 10.1186/s12864-014-1200-y (PMC4311427; doi:10.1186/s12864-014-1200-y)
Supplement: Additional file 10: — S11-nucleotide sequences of OBPs. [file 12864_2014_1200_MOESM10_ESM.pdf]

>Cmeg1105458_c0
AAAAGCAATAGCAGCAGCAAAAAGACAACAGCAGTTATATGATGCATGCAAAGTAGAAATGTTGCAAGTTGTGGGGGAAAAAGAGTA
GTTAAGGAATCAGCTGCAGTAGACAGTTTAGAAAAGAGTGTAGAATTAAAACGGAATTATGATGTAGTAGACTAACAGGCTATGATCT
CTCCTTAAAAAAAATAAGAAAATTGAAGTAAAAGATTGCTGTTCTATATAAAAGGTGAAGCTA
>Cmeg12669_c0
AATTTCAAAGAGTATAAACAAATCTTTTGATCAACAATGAACAACTTAATATACTTATTAATTTGCTCACTATTAGGGACAACTATAGCA
GCGCCGCCCAGTTGGTATCCTAATAATGCCAAGGAATTGACAAAAAAATGTATGGAGGATAATAATATAACACCTGAAATGGCAAAGA
GATATGGTTCCCTATATGATAATCCTATTAGACGTACCATTATGCTGTGTAATGTTAAATCTTCGAACATTTATAGTGAAGAAACAGGAT
TTTATGTAGATCGAATGGCTTATATGTTCTATGAACCTGCTAGTTATGTAGGGGCTAGAAAAGCGATATTACAAAATTGTGTTGATGAA
CATAAAGATTTAAGCTCCCATGCTGAAATAGCTTTTAATACGGTTAAGTGTGTTCTAGACACGGATGATAAGGAGATAAGCAATTTGAA
TAAAAATTAGAAATAATTGGTATACATTTTGTAATAAA
>Cmeg16201_c0
CGTGAAACAAACAAAATAGTTTTTGCAAAAAATGGGAAAACTTTTTGTAGTTTTCACAATTCTTGGTATTTTTGCAGCTGTTTCAGTTAAA
GGATTTGATAAGGAGACAGCTAAGGCTATTTTAAGAGAAAAGGCAGAAATGTGTAAAGAAAAAGTTGGTGCTAGTGAATCTGATATTA
CAAATATAATAGACCAAAAGCCATCATCTACTAAAGAAGGTAAATGTTTACGCCACTGTATTATGGAACATTTTAAAGTGATGGATGAA
AATGGTAATTTCGATAAGGATATTGCAATGACACATGTAAGGTTTTTAACAGATGGTGATGAAGATAAAATAAATACTGCTTCGGAAAT
AATAGACCATTGTACTGGCACTGTAGCTGAGGACGATAAATGTGAAGTTGCTGAACAGTATGATATATGTTTTATGGAACAAGCTAAAT
CTCATAATATTGATGATATATTTGAGATGTAAAACAGAAATATAATGAAATTTGATGTAAACTTAGAACATTGATTAGG
>Cmeg618005_c0
GGGTGGGTCCATAAATCTAAAGGACCGAGGGAAGTAGGATATGCGGGCAGCTAAATATATGGTTAGTGTAGTGTGGACTCTGACAAC
ATGGTCATGAATTTGGGACGGCATATTTTATGCGGGACTAGTAGGAGTTCAATCTTTTGCTACATCCCGATCTCAGTAGTGCTAGAACC
ACTCTCGTATTTCCCAGCAGATATTCTTCTTCTTCAATAGGCGGTGAGCGACCTCCAAGTAC
>Cmeg20107_c0
ACTTGTTTCTTTGAAAAAAATCGGTGTGTTAAAGAATAGCGTTGTACAGGAATCGGTAGTTTTGGAGAAATTATCATCTATTTTTGGTGAG
GAGAAAATTAAAGCTTTCCTAAAGAAATGTAAAAATGTCAAAGGCTCTGAACGTTGTGATACGGGTTTCAAGCTCTATCAGTGTTTCGA
AAAGGCTTGAGATGAATAGGATGAATCTAAAACGTTTTCTTTTCTATGGAAAAAATTGACCTAAAGCTGCCTTTTTCTGTATGTAACTAT
AGGGAAGGGCAGCATAAAGGCAATATTTTGAACAGAAAAGAATTTTATAATTTTTAAACAAAACATTTTAAATTAAGTTATAGGTGCCT
ATATATATTTAAATAAAATATATTTTCA
>Cmeg21243_c0
TTTGAATTTATTATTCTAACAAAATAAACCAAAAATTAACAAAAAATCAAACAAAATGAAAGTTTATATTTTATTAATAACTGTAATTTTG
GCTGTGGCACAGGTTAAGTGTGATTTGAAAGAGGATATACGCCAGGCCAATATGGCCTGTCTTGAAGAGACCAAAGCCAATCAAGATG
AGTTGAAAAGTTTCTTTAAGGGCGAATTAAAAGGAGAACCTAAAGAGGCTCTTAAATGTCACTTGAAATGCTTTATGGAAAAACAAGG
ACAATGGAAAAATGGTGCTTTTGATGAAAAATCTGCCATAAAATATTTGCAAAATATACCAGCCTTAAAGGATCATCAGGATGCCATTA
ATAAGGCTATGAATGATTGTAAGACTCAAAAGGGTTCCAATGAATGTGATACTGCTTATTTGATTATGAAATGTTTGGGTGAACATAAA
GCTTCCATGATGTGAACAATTTAAGAAAAAAACTTCAGCAAAAAGGATCAAAACTATGTGAGAATTTTAAATTTTATTATACAAAAAAA
AGTTTTAAATAAAAAATTGGTTATGGAA
>Cmeg21269_c0
AAAAAAAAAAAAAAAAAAAAAACAATAAAAAAATAAATTAATTAAATGCATTAATAATATTGTTAAAAAAATACAAAATAAAACTAGAT
TTTAAAATGCTACTGCAAATTAAGTTTGTTATAAAAAGCCCGACTTAAATATAGATCTAGCACAGTCAACAAACAGAAACTTTAAGAAAC
AAACACAAAACTCTAGAAATCTAGACAACATGAAAGCTTTTATTACTTTAGCTGTTGTTTGCTTAATTGCTGGTGCTTTTGCTCACCTTGA
ATTGAGCGATGAACAAAAAGCCAAAGTCAAGGTTCATTTTGATGAATGTGTTAAAGAAGAAAAAGTTTCCGAAGCAGAAGCCACCAAA
TTGCGCAACAAGGACTTTGCTAATCCCACACCAGCTATGAAATGTTTCGGTACCTGTTTCTTTGAAAAACTTGGCACCTTGAAGGATGGT
GTTGTCCAAGAAGCTGTTGTTTTAGAAAAATTGGCTCCTCACTTTGGTGAAGAAAAAGTTAAGACTGTTTTGGACAAGTGCAAGGATAT
TAAGGGTGCTGATCGTTGTGATACTGGTTTCAAAATCTATGAATGTTTCGAAAAAGCTAAAGCTGAATTGGGTCACTAAAAAGTGAGAA
ACAATTTGGCGTATATAGTATTAAGAAAATATGTAAATGTCTGTAAATAAAAGAAATATTTTAAAATTTATTTTAAAAAAAAAAAAAAAA
AA
>Cmeg21549_c0
GGGAACAGTTTTAAATTAATATTGTAACAATTGTATAACGTTAAATAAATTGAAGCAGTTTGTCATAAAAAAATGGGAAAACTTTTTGTAG
CTTTGGCAATTCTATGTGTTTTTGGATCCGTTTTAGTACAAGGATTTGATAAAGAAGCAGCTAAGGCTGCATTGAAGGAAAAGGCACAA

ATTTGTAAAGGAGAAGTTGGTGCTAGTGATTCCGATATAGCAGAATTAACAGAGAAAAAACCAGCATCTACTAAAGAAGGCAAATGTT
TACGTTCATGTATTATGAAAAAATGCAAAGTTATGAATGATAATGGTAAATTCGATAAGGATACAGCCATGTCACATGCTAAAATGTAT
ACAGACGGTGACGAAGATAAAATGAAAATTGCTACTGAAATAATTGATCATTGCAGTGTCATTGATGTCGACGATGATGAATGTGAAG
CTGCTGAACAGTATGGA
>Cmeg21654_c0
AACAGTCTTCGAACTAAACAAACGGAAATCAATATGAAAAGGGTTTTAATTGTTTTATTTATAATTTGCTTTTTCCAATATATTTCAGCAA
CGAGTATTGCCAATGAATTTAGATCAGCTGATTTTGAGTGCAAAAAAGAACTAAAAATCTCCAAAGAAGATGCCAGTAAATTATTACAA
TCAATTGAGAATCCTACTCTAGAGACGAAACTATATTTCAATTGTTTCCTGGAAAAATTAGGCTTTTTGAAAGATAATATTTTGCAAGAA
AACTATATTCTAAGCAAGACCGATGAATACAGTGATGCTGGATATTTAAGAAAATTTGTTAGCAAATGCAAACATATTAAAGGTGAAGA
TCAATATGAAACTGCAGTACAATTGGGAATTTGTTTTCATAATACCAACAGAAAAGAAGAGTTTTAAATCTAAACTTTCAACTTAAGACT
AAACATAAGAATATTGTACAAAT
>Cmeg22689_c0
AAAGTTTTGCAAAAATGTTTAAATTTACTTATAATTTAATAATTTTTTGTTTAATTGGTATAGTAATAGTTAAAGCATTGGAAGAAGGGG
ATATGAAGGCTATGGTTTTAAAAGTGGGAGAAGGGTGTAAAGCAGAGACGGGTGCAAGTGATTCCGATTTACAGGAACTGTTGGATA
GTAAATTTGCTTCAACCAAGGAAGGTAAATGTTTACGATTTTGTGTTATGAAGAAAATTGGACAAATAGATGACAATGGTAAACTACGT
AAGTTGCCTTACACAGAATACATGACAGAGAAGATAAAAACTGGAGTTGAAATAATAAATCAATGTACTGACATTGAAGTTGATAATG
ATCCTTGTGTAGCTATTGAACAGTATGAACAGTG
>Cmeg23484_c0
TTTTTTTTTTTTTTTCTTTTATAGAGTTTGCGCTTTTTATTAATTATAAATTAACGAAAGTTATTTTAACACTTTAATATAAAAAAATCATAT
TATAATAAAAAAAATGACGATTGCGGGACGTAAGAGATGATTCATTCTTTCATTTAACCATTTTATTCTTCCTTTTCAATGTAGTATTATCTT
CTTTGCTACCAAAACATAGAGATGATCGTCAATGTTTTTTTGGTAACATTTGTATTTTGTAATTGTGTGGCTGTCTACATTTAAATTTCTTT
CTGTTGTTTTGTTCAGATGTTATTGTTGTTGATATTTTTCCGGCTGGTAATTAGATATGTAAAAAAAGTTTTAAAGTAGTAATAGTAATTA
TCCTCCCCCTAAGATAATTTAGTTTTTCTTCAAGCTTTCCATAACCAAAGGTAAATTCTTGCTAATGAAACACTTGAAGCCACGGAAAGCC
CAGGCGCATGAATCGGATTTTTGTTCATTCTTATCGGCACACTTTTCAACATCAGCCTTAACTTCGTCTTTCTTTTCCTTGCCATGACCGAG
TTGAGCTACCAAGTTGTCGTTTTTGAAACCATCCTTAGCATCGAACAATTCGAATTTCTCGAAAATGCATTCAATGTAACAGCGTGTTACT
TCATCATCGGGAAATTCGAATTTCTTGTATTTCTCAATCAATTCGGCGGAGACTTTTTTGGCTTCCATACATTCTTTGCGGGCCTTAACAA
GATCTTCTTGGGTGCGTAATTTGTATTCGGCAGAGACCAAAGCAAAGAGAGCGATTAAGATTGCTAATGTTGCCTTCATTTTGTTAGTG
TTTAAAAAAATATTTAGAACAAAATCTACTTTGAATAAATCACAAAAAAATACTTTGTACGTTGGGTTTTGATGTTTGTCTAAGCTTGATC
CGTTTAAAACTGATGCCGAATGTCACAAATCGCAGAGATTTTATAGTCA
>Cmeg23600_c0
AGGTTTAAAACGGTTGTGTTTTCATTTAAAGAAAAATAATTTGAAAATATTTGAAAAAAAAAGAAAAATGTTGAAATCTGTTATATCCTT
GTTATTAATAGCAACATTGCAATCTACTTTGGCTACCATAATTGATTGTCAAAGGCCTCCTCAATTAGTGGATCCTGCTAAATGTTGTTTG
GATGGTGGTCGAGATGAGGTAACAGAATATTGTGCTCAAAAAATGGGTATTACCGGTAGACCCAGTGATGCTCCCCCTACAGTAGAAA
CAGCGACATGCCTGGCCGAATGCATTTTAACCGAATCCAAATACATGCAAAAACCCGAAACCCTAGAAATTGGCATTATCAAATCCGAT
CTTTATAACAAATTCTCCAATGATACAATTTATGCCGAAACCATGGCTGAAGCTTTTGCCAAATGTCAACCTACAGCTCAAAGAAAAATG
AAAGCTTTCAAGCAAATACCTCTTGGAAATGTGGCTTTGCAAAGAGGATGCAGTCCTTTTGCTGGTATGGTTTTGGGCTGTACTTATATG
GAATATTTCAAAAATTGTCCGGCACATCGATGGACCGAAAATGCTGAGTGTAGTTTAGCGAAACAATTTGTTACACAGTGTTCTTTGGG
AGCTTAAATGA
>Cmeg23854_c0
TATCACAACGATCTTCACCCTTAACATCTTTACATTTCTCCAAAATAGCTTTGGTCTTTTCCTCTCCCAAAATGGAACCTAATTTAGCCAAA
ACAACATCTTCTTGTACCACACTATCTTTCAAAGTGCCCACCTTTTCAAAGAAACAAGTACCAAAACATTTCAAATTCATTGAGGGATTAG
AAAAATCTTTTTTGTCCAATTTGGCAGCTTCCTCTTCGGTAACATGTTCTTGTTGGATACATTCTTGGAAATATTCTTTAGCTTTGGCTTTT
TGTTCATCATTCAATTCCAAGGGTGATGTACAGGCTTGAGCAATTAAACAAAAAATAGCTAAAGTAATAATTAGTTTCATTTTAAGTTTA
TCTCGAAATGTGTTTGTTTTAGGGCAGTTAA
>Cmeg24434_c0
TTCTCAATTATAAAAATATTTCTATTTAATTTTCTTTTAATTTTTCATAATGTTTGTATAATTTTTATAACAAAATTACAACTGATCACATTTT
CAAATTATCACTAACCACTCATAAGGGTCAAATTTTAGTTGGTACCCAAAATTTTACGGACAGCATGACGATTGCTCTTAATCCAGCAAG
TGAAAACACGGTGAGCAAATGTACAAACATCCGATTCAGATTCATTGTGATCAATACATTTTTCCAAACCATGTTGAACACTGCTTAAGA
ATTTGGGATTACCATGAGTGAATTGAGCAATGATGGCCTTTTCATCGTATCCCTTTTTAGGATTGAAAATGCCCATTTTCTCAACCCAACA

TTTAATATAACATTTGGTATTTTTATGTTCAGGGAATTTATAGTCAAGGAATTGTTCATAGACATCATCAGGTACATTGTATTCCTCACGA
CATTCTTCGTGGTTTCTTATGGCATCCTCGCTAGTGCGAATTTCAAATTTGGCTGAAACCACAGCCAACATAACTAAAATTAAAGCAACT
TGCAATTTCATTTTCGATTTCAATAATGTCTCTTAGATCAGTGATGTAGATTTCTTGAAGGTTTATTCAAGACTCTCAGG
>Cmeg248939_c0
TCAATCACTTCTTTCAAACCGTTAAATAGTTATCTGCAATAAATTATGGGACATTTACAAAAACTAATATTATTCTGCTTATTTACCTTTTC
GTGGTCTGCCAATCCTTCTTTAAACCGATATGAAGCAGCCAGAAAATTTCAATTGATACTGGAGGAATGTAGAGAAGAAGTTGCTGCTA
CAACAGCTGATATTCAGGAATTATTGAATAGAAAACCTGCTTCCACTATGAGCGGAAAATGTTTTCGTTCCTGTCTTATGAAAAAATACA
ATGTTATGGATTCAGATGGTAAATTTGATAAGGAAGCAGCTTTAGATGAAGCTCGTAAATTAACACGTGGAGATGTTTCTAAAATGCAA
TTGGCTGAAAATTTAACAAATGCCTGCAGTGATATTGAAGTTTCCTCTGATCATTGTGAAGCAGCTGCTGATTACGGTGAATGTTTTAGG
GAACAAATTAAATCTTTGAATTTAGCTAAATCTTAAAGTTATTTTAAATATAAAATTTTGAAATCGATTACGG
>Cmeg24919_c0
TGGTAGTGGCTTTTTTTGAATTTTGTTCTTTTTTTCTTCTTTTGATTTTTTTCTTTTTTTTTTTTTTCTTTAAATTTTATTTTATTCAACTTCAGA
TCTATTCCTTTTTTAATAACTCAATATGTGTGTCTTTAATTTTTTGCAAACATTCAAATAGTTTGAATGCAGTTTCAGTTTGATTATTTTCTT
TGATATGTTTGCACTCATTAACAAATGTTTTTAGATACTCAGCGTCCTTATAATCTTCTGATACTTCCAAAATAACATTCTCCTGTAAAATA
TTATCCTTTAAAAATCCTTCTTTCTCAAATACACAAGCCGTATATTGTTTCATAGCTGGCGTAGGATTATCAACAGGAAGATGTTGAAGTT
TTGTATATTCTTCTTCTGAAAGTTGTTGTTCTTCTTTACAGTCCTTACTGGCAAGTTTCCATTCTTTTAGAAAATCAGATGTATCAACTTTTC
TAAAACATAAGCCCATTTTATAGGCTGTATCATCATGATCAATGCCTTTTGTGTGTCGACAAACACGTACACAATGTTTAAAAAAATTCG
GATACTTAAAATCTTTGGATCTTTTTAACATAATATCTTCCTGCATAACATTATCCTTAATGAAGCCTACTTTTCCCAAAAAACAACCAGCA
AACATTTTTAATCCAGGTTTTGGATTTGTCAGATCATTATCTCCGAATACTTCATTTCTAGTTAGATGTTTTTCTTTTAAACAATCATCTTCG
GCAGCTTTAAATTCTTCATCAAGGCTCTCTGACAAAGAATTGCCCAGCAAACAGAATAATGCTAAAGTTATAAAAATTTTCATTTTAAGA
TTAAATCTATTAAAGATCGTTTAAACACTGAATCTTTTATTTCAATTTGTTAGCTTTTTATAGTGAATTGTTTTAGAAAATTTTTTATATTG
>Cmeg24940_c0
GATACCTATAATTAATTATAATTTAATAATTAAAGTTTGAACACTGATTAATCCATTTCTTCTCAAGCCTCTATATAACACTTTATAAATTT
ATACATCGTTTCACAGCGGTCGGAGTCTTTAATATGTCTACATGTTTTTATGGCATTTTCAACTCTTTCTTTGCCAAAAATTGGACCCACTT
TCTCTAAAACTAAATCTTCCTGTATAACACCATCCTTCCAAGTACCGATTTTTTCAAAAAAACAAGAACCAAAACATTTTACGGCTGGCCT
TGGTTTAGTCGTAACATTGTTAAGCAATTCAGTTCCTTCCTCCTCACTAACCCTCTCTTGTATTAAACATACATCAAAGGGAATCCTATTCT
TGGCCTTTAGTATTTCAACTAATTGAGCGTGATTTACAGCAAAAACATTAGCAATTAAGCAAATAACAACCAATAATATAAAGATTCTCA
TCATATATGTTTACGAAAGATTTTAGGTTT
>Cmeg24941_c0
AACTTGCAACAATGAATACAGTTTTCCTAAGTCATTTACTTCTTGTGGCAGTGTTGCTACTCAGCTTTAAACAACACACGGATGCTGCCCC
AAACGCTGATGCTGCCAAAAAAGCCGCAGAGGAATGCATAAAGGAAGGTGGTTTAAATGCTGAAGATTCTAAACGCATTATGGCTAAT
GAATTATTTAGTCCCAAATACGAAACCGCTTCGGATAAACTACAATGTTTCTTATTGTGTTACTATAAGAAAATTGGCATTATCGATGCT
ATCGGCAAACAGAAAGCTGATGTTTTCATGGGCTATTTAGAACATAGATTTTCGGATAAAAAGGATAAAATTAAACCGGCTTTGGCTAA
ATGTTCCACGGTTAAGGCTACTAATCCTTGTGAAGCTGTTTATGCTTTTGAGGCATGTGTTTTGAAAAATATAAATTAAAATTCTTGTTTT
AATTATTTAAAGGCAAAATTTATATTTATTTGATTGTGCATTTTTCTAATAAATTTGAGAAATA
>Cmeg25217_c0
TTTTGGTACTTGTTTCTTTGAAAAAATCGGTACCTTAAAGGATGGTGTTGTTCTAGAAGCTGCAGTTTTGGAAAAGTTTGCTCCTGCTTAT
GGTGAGGAAAAAGTCAACGCTGCCTTGACCAAATGCAGAGGTGTTAAGGGTTCAGATCGTTGTGATAATGGCTTCAAAATACTCGAAT
GTTTTGAAAAGGCTAAAGATGAAGTGGGAATTGTTTAATAATCAAAATAATCAACATTCAATTCAATAAAATTTTCGTTTAAAACTAAAA
AAAAAAAAAAA
>Cmeg25756_c0
TTGAATCAGTAAACTAACAAACTATCAAGGAAATACAATTCATAATACAATATGAAAGTTTTTATTGTATTAGGTCTAATCTTTTTACTGG
GCAATGCTAAGGCTGCGACCCTACAAGATGAATTAAATGCTGCTGAAGATACTTGTATCAAAGACAATAAACTCAATCGAGATGAAGT
ATTCGGAGATTACACGAAACCAACCGATTCTTTAAAAATTTTTGCCGGTTGTCTTTTAGGCAAAGTGGGCTTCGTAAAGAATGGCGTAG
TACAGAAAGAAGTTATATTGGAAAAAACAAAACTTACTAAATACCCGAAATACTTTAAACATGCTGCTCGTATTTGCAGTCACATAACG
GGTACAGATCACAATGATGTAGCTTACAACTTGGGTATGTGCTTTAAAAAGGTTAATGGTGAGATATTCCTACTTGAATGGAAAAACTC
TGGTATGGAATGCAAAGAAGAAGAACAACTCTCCGATGAGGATTATACAAAACTTCAAAATCTTCCCATTGAAAATCCCACTTTGGCAA
TGAAACGTTATACACATTGTATTTTAACAAAACAATCTTTTCTTAAGGATAATGTTTTGCAGGAAAGTGTTATATTGGAAGTAGCAGAAG

ATTACGAAGATGCAGATTTTCTAAAAACGTTTGTAAATCAATGTAAACAAATCAAGGAGGATGATCAAAATGATACGGCTATTAAATTA
TATGTTTGTATGTTAAAAAATAATAAAGCATAGATTAAGAGATTTTATTATCGTGAAATGAATAGAAAA
>Cmeg26369_c1
AAATTTTCTATAATAACCATTTAAAGTTTTAAAGGTTCACTAACTTAGGATTAAAATTTAAGTTTTTTAAATTTTCAAAATTTATAACGATT
TGTATTATATTTATTAGTTTTTGCATAATTTGTTTTATTACATTTCGACTATTTTAACAATAACTTTTATTTAATAACTTTTCAACAAAATTTA
ATGACAAGTTGTGAGATGAAAAAAATCAACTTATGTTGCCTTGTATTTTCCTTAATTTTTTCTTTAATATTTAAAACAGAAAATTATAAAA
TTTATTTAACTACCCAA
>Cmeg26380_c0
TAAAATGAAAATAGCTCATGAAATAATTGATGCCTGTGTGGGTATTACTGTTCCTGATGATCATTGTGAAGCTGCCGAAGAGTATGGTA
AATGTTTTAAGAAGGAATATCTAGCTCATGGTCTTAAAGAAATTGAAATTGATTTTTAAATCATAAGAAGACAAGGTTGAAAGTTTGAT
GTTTATTGTTTTGGAGTTTAAAAAGTATTATAATATTTTGTTGAATTAAATTGGCGTTGTATTTGTAGTTTAAATATTCCAGACATAATTA
CGTTATAACTCATTTAAAATTGTAATTGTTTAATAAATATTTTAAAAATGGCGTAAGTTCATTTACCTTAGAATCAA
>Cmeg26380_c1
AACAATTAATTAATATTGTTAATCGAAAAAAAAACTATTAAGGAAAAAATGGCAAAAATTTTTGTGACTTTAACTATTTTATGTGTTTTCG
GTGCTGTTTTGGTTAAAGGTTTTGATAAGAAAAAAGCTATCGCCGATTTTATGACTAGAATTGATGAATGTAAAGTTGAAGTTGGAGCC
AAGGATACTGATGTTGAGGAATTACTGGAGAAAAAATCAGCCTCATCCATGGAAGGCAAATGTTTACGTTCATGTCTAATGAAGAAAT
ATGAAATGATGGATAGTAATGGAAAAATTGTTAAAGATAAAGTCGTAACTGATGCTGAAAAATATACTGATGGTGATGCCGATAAAAT
GAAAATAGCTCATGAA
>Cmeg26644_c0
GTCGGACAATCTTTAAGTCATTTTAAAGCAACCGTTATATTTATGTGTACATAAGATCTTTGAAATTTACAGCTGTAGCTTTTTCATTAAA
ACAGTGTTCATATTGATCCATGGCTTCGCAAGGATCATTGTCAACATCAATATCAACACATTGATTCATTATCTCACCTCCAGTTTTTGTA
TTCTCCGACAAGGATTTTAATATAGGTAATGTCTTAAGTTTACCTTTGTCATCCATTTGTCCCATTTTCTTCATGACACAAAAACGTAAAC
ATTTACCTTCCTTGGTTCTTGCAAATTTATTATCCAGAACATCCTCTATATCAGAATCCTTTGCACCAGATTCCGGTTTACAACCAGTAGCA
ATTGATTTAATTATAGTCTTAGTTTCCTCTTCACTCAATCCTTTAACTACAACAATTCCAATTAAACAAAAAATTAACAAATTTAAAAGAAA
TTTTGACATTTTTTAAATATTAGGCGTTAAGTAAAATGTAATTCAAA
>Cmeg26709_c0
AACAATTAAACAATTTTAAATTTAAGAGAATTTAGAAAATAATAACTAATAAAATGGCTAAAATTTTGTTAACTTTAACAATTGTCTGTGT
TTTTGGTGCCGTTTTGGTCAAAGGATTTGATAAGGAAAAAGCTATAGCTGGGTTTATGGCTAAAATGGACGATTGTAAAGCTGAAGTT
GGAGCTAAGGATACTGATGTTGAGGAATTAGTAGGTAAAAAACCAGCCTCAACTATGGAGGGCAAATGTTTACGTTCCTGCCTTATGA
AGAGATACGAAGTGATGGATGGCAATGGAAAATTTGTCAAAGACGTTGCCATATCCCATGCCGAAAAATATACTGATGGCGATGAGG
ATAAAATGAAAATAGCTCATGAAATCATTGATGCCTGCAGTGGTTTAGATGTCTCGGATGATCATTGTGAAGCTGCTGAACAGTATGGA
AAATGTTTTAAGGATGAATCTATAGCTCATGGTCTTAAAGAAAAATTGAATTTCTAAAATATGTTTATGGAAAGGGAATAAACGAGAAC
GGAATTAATTGTTGATGATGATTGTTATTGGCTTTGATTTAGATTAGTATTTTAAGTTTATTATGTAAATTTTTTGTTGAAATATATGCATT
GACAATTGCAAG
>Cmeg27265_c0
AATAGTGTTGAAATTTAATAGTGAAAATAAACTAAATAGAGAAAACGACAAATTAAAGTGACACTTTCAAAGAAAGTGTTTTGAGAAAT
AAATATGAATTTATTGCCAAAATTGTTAATATGTTTTAGCATTATCGTTCTAACATATGCTGATGATGATGGAATGAGTGTCAAAGATAT
TGCAGAAGCTTTAATGTCATTTGGTGAAGATTGTGAACCCAAAGCAGAAGAAGAACACATCATTGAAGTGGTGAAAAATGTCAAAGAT
GCTCAATATACTTCAAAATGCTTTAGACATTGTTTAATGTCACAATTTGAATTGATAGCAGAGGGTTCCACAACAATGGATAAAGAAAA
AACCGTTGATATGATGGGTTCTATGTTTTCTGATCGCAAAGATGATCTATCACAAATTATTGATGAGTGTAATACTAAAAATGAAGCCAT
TACAGAGAAATGTGAAAATGCTCATGCCCATGGTATGTGCATGTTGGACCTAATGAAAGAACGTGGTTTTGATATACCTGATCTTAAAG
ATGAATAATGACTTGACCGCCATCCTCGCCCCCAATATACCTGATAATGATTCGTAATACTTGCCTATAACATTCTTCGGTTTTCCGGTTT
ATATTTAAACTTTTTTAATATAATTTTATTTACACAGCATTAAATCATACGGTACCTCAAAGTGGTGTTTATTAAA
>Cmeg27557_c0
TTATATAATTTTTATTTATTAATTTTTAAAATTAATTATTTCTAAGTTACACTAAAGGTTCTAAGTCACATTATTAATTTTAAACCATTGAAG
TAACTAAGAGTTATACACTTATTTTTATTGATTTTCAAAAGAAAACTTCATAAATTCACAAAAAAACTTAAATAAATTACAACTTATTTTG
CACATTTTTAACACCTCTAGACATTAAGAAGCAAATAAATTCTCTATACATCCAGGCACAGGAATTGCGGCTACGTCTTTGACCCATAGC
CAAACAATTTGAAATATTAGCATTTTCTTCGGGAAAACCTAATTTCTCCAACATATTGGCGGTATTCCATTTGCGCAAACGATGATTGAA
CAATTGTAATTTTTCCACAAAACAACGCGTGTAGCAGGGAATTGGTTCTTTGACGGGATAAGAGCTATACTCACCAAACTTTTGCCATTC

GAATTGATCGAATTTATGTAAACAATCTTTCATGGCCAATTTGGCATCGGCATTTAACCCTTGCATACTTTCAATAACCACAAATGGATCA
TTTTCAAGATTAACCATACAATGAAAACCATTATAGGCAATTTCACAAGCATCATTGCCTTCAATTAATTTATTTTTACAGGCTTCATAGA
TAACATTGCCAAAATGTTTAATAACATTTGGTCCATGAAATCCAGTAGTTTCATTATAAAAATCAAACATTTTATTCAAATAACAGTTGGT
GAAACAGGGAATTTCCTCATAGGTATCAGACCATTGACGAAAACGTTGTAAACGTTGTGCATTTTCTAAAGTAGGTCCACCAAAACGAT
CTAAACATTCTGCTATGGCTTTACCTTCCTTAGGATCAAGATTTTTATCACAATTTCCCACCACTAATGTAACGAGACATAAGTTTAAAAA
TATCGTATTAAAAGTTTTCATTTTGCTCCGATTTATAAGAGATTCTAATGTTGTCATTAAGAACTATAATTATAAACTGACATTGTTATGCT
AAAGTAAAAACTATTTTTTGGTGCTTTTAAATTATTATTTTTTTCTTGTTATTATTTTAGCAAATCATATCACTTTGATAATGAATTAACAAT
GTATGTAGTATATGCTACCCTTTTCAAATTCACAAATATGGAACTGATAAGAGATAGA
>Cmeg27729_c0
TTTTGGAAAATGTTTGATATATTGCAAAATGATTACGTTTTATTTAAATTTAAATTACACTGAAATTTCTTGAAACTTTTCAATAAAATATA
GATATATACCTAAAAATAAAAATGTTTAAAAAATTTTAATTTCTTCCATACAAATTCTGAAGTATTCTCAGGCCTCCAGGAAACATTTAAT
GATTTTGAACACTGTATCACAACGATCAGAACCTTTAACATTGTTGCATTTTGTTAGGGCAGTTCTTATCTTCTCCTCGCCAAAAATTGGT
ACTAATTTCTCCAATATAACTTTCTCTTGAAGAGTACCATTTGTCCAAGTGCCGACCCTTTCAACATAACAAGCTCCAAAACATTTTATGTT
ACGAGATGGATCAGTCAACTCATTACTGAACAGTTTAGTTACATCCTCTTCAGAGATATTTTCTTGCTTAATACAAACATCTATATGAGCT
CTGCTATGAAGCTTTAAAAGCTCTGTTGTACGTGCAAATTTGTTATTTTGAGCAAAAGCTCCAGCAATTAGGCACAGGACGGCTAAAGT
TATAAAAGATTTCATATTTTCTTTAGATTTTAAGAATTTTATTTAATACGTTATGTTTTAGGTTT
>Cmeg28108_c1
AAAAGACAAAAAGACTTCTTATCAACATTAAACTCGGGGATTTTACGTTGATTTTAATTTAAATTAAAATTGACAAAAACGAAAACAAAA
GATTTAGTTTTGTAATACTATAACAGAGGAAAAGAAATCTTAATATGAAAATTTTTATAGTTTTGTTTATCTTCTATTTACTGGACTATTCT
TTGGCAGTGTCCTTTTATGAAGAGAATGAAATTGCCGAAAACGATTGTTTAAGAGAGCAACAGCTAACCGCCGAAGATTTCTACCGAAT
ATCAAGAGCACAGCGAACAATTTTATTTAAATGTATTTATGAAAAATTGGGATATATAAAAGATGGCATTATCCAGACAAATGTTATATT
GGAAAAATCCAAAAATTCAGCAATAGCGAACAAATATAAATATGGTGTTCGCCTTTGCGAATACATAATATCTAATGATGAGCATGATT
TGGCCTATAAACTAAGTGAATGTTTTAAAAAAACTAATCCAGATTATTTCAAAGAAATTGGAATATCTTAGACTATGAATGTAAAGATA
AAGTGAAAATTTCTAAATATGATTATAAAAGACTACAATATTTTCCCTTAGAAAATATTACTGAACCCATGAAATTATATACTAAATGCAT
GATGAAAACACTTGGTTTTCTAAAATACAATAATCTGCAGGAAAAAGTTGTATTGGAAGTATCAGAAGACTTTGAAGATGCAAAATATC
TAAAAGCGTTTTTGAAAGAATGTAAAGGTATTAAGGAATATAATGAAAATGATACATCATTTAAATTATATGAGTGTTTGGAAAATGTT
AAAGAAAAATATGTTCTGAGCACCGAAGATCAATATAAATTGGCACAAAAATCTTGTCTCCAAGAAAAACAACTCTCCCCCCATGAAGT
CTACCATCAACAGTCCCCTTATAAACAGATGTTTGCTAATTGTGTTTTTGAAAAAATGGGTT
>Cmeg28108_c0
TATGAAAATGAAAAATCCAACTCCAGCTGTCAAATATTATGCTGCTTGTATTTTTAAAAAACTTGGCTTTCTAAAGGCTAATACTTTGCAG
AAAAATGTCGTTTTCAAGGCATTAGAGGACTACAGAGATGCGGAATATTTAAAAATTTTCTTAAGTAAATGTAAACACATCAAAGAAAA
AGAACATATCGATACGGCTTATAAATTATATCAGTGTATGGATAAAATTATACACACAGAGAAAAAAGATGATAGAGAATGATCACAG
CAATAACAAAATGATTGTGGAGACATTTTTTTATATTCAAGAAAACTTTTTTCACTATAATAACGTTTTTGATTGAGT
>Cmeg28341_c0
TAAAAGCTAAAGAACACTAATATCTTTGTGGAGTTTATTGAATTTCGAAAAAAATGGCTTATGGATTTTTGGTTCTAACAATTTTAGCCG
CTTTAGGGCATACTTGTCTAGCAGCTGGAGTAGATTGTAAAAAAATGCCACCCAAAGTTGATCCAGCCTCATGTTGTCCTATACCTGAAC
TAATAACCGAGGAAAATAAAGAAGCATGTAAAGAATTTTTAATGGAACCTAAACCATCGTTTGTACCTATGGGCAATGAAAAACCAATT
GGTACTGCTAATAAAGCAGCTCCTACACCAGCAAGTCATCGTTCTGATCATTATCACAGGATTAGAAATGAACCACTTATGCATTTGTGC
TTTATGAATTGTGCCCTTAATGATACCGGTATTCTAACAAATGCTAAACTTAACTCAGCCGCTCTTACTACACAACTGAAAAAGGTCTTAA
ATGATACCCCCGATCTGATACCGGTCTTGGAAACTTCTTTTAAAACCTGTTCAGCAATGGGTGAAAAATTCCATCAGAAAATGCAAGAA
AGAATGAAAAAGAGAAAAATGTCTACAACTCCAGCCACCGCCACTAAAGATCGTATGCTTAGACCATTACGTTGTCCACCAATCGCCAG
TCATATGATGGCTTGTGTTTTTATGGAAACATTTATGAAATGTCCTGCCTCCGTATGGACCAAGACTAATGAATGCAATGAATTACGTGA
TCATATGCTAAATTGTCAACCAAAATATAGTATGGAAGAATCTAGTGAAGAGGATGCTATGTAAATTATATAAATTTACTCTAAACCAAA
AGTGAA

>Cmeg28677_c0
GAAAACTTCGAAAATGGCTGCTACAAAATTGAAACTTTTCCGACTAGTGCTAATAGTGGCAGGGTTTGCTGCTACTAAAGCCGAAGTGG
ATTGTAAAAAGCATCATCCTCATGTGGATATGAAAAGCTGTTGTGAAATATCAAATTTCGTATCAGACGAAATTAAAGAGAAATGTGAA

GTGGAAGGTCCCCCTGGACCACCTGGAGTTGGTACCCCTGGTGATGTTCCCCCAGGACCACCTGGAGGTCATGGACCTCATGGTCATC
ATCATGGTGGACCACATGGACCTCATCCTTGTTTCGTAGCATGTGCTCTCAATGAAACGGGAATATTGATTGATGATGAATTACAAGAA
GATAATTTACAAACCTATTTAAATGGTGTTTTTGATGATGGAGATAAAGTAGAATTTTTAATGGAAAAATTCAAATATTGCGATGAAAA
ACGTAAAAATTCTGGTGAAAAACAGCATCATGGACATGGTCCACCACATCATCATCACCATAATTGTGGTGGTCCCAAACATGGTAAAA
TGTTAGTTGGCTGTGTTTTTATGGAAACTTTCAAGGAATGTCCGGATAATGCTTGGTCAAATACAGATGAATGCAATGAGGCTCGTGAT
CATTTCATTCAATGTCCACATTTTGGACCTGGTGAAAAACCACAGGAAGAAGATGTAGAAGCTATTTAAACTATTTTATTAAAAATGTTA
TTAAAAGAGATCATTAATATCTAATAAAGGCA
>Cmeg28701_c0
TTACAACTTTGTTTTATAAATTTCCAAAATTTATTAAGGAAATATTGAAAATAATTTATTTATAAAAATTACATTTCTAATTGTGTTAACAA
AATGATTTTAAAACAAAATTTAATTTTCCTAATTGGAATTGTAGTGTGCATTTGTAAAATAACGGAAGTTTTTGGCGGTGCTACTGAAGA
ACAAATGTGGGCTGCTGGTAATCTAATGAGAGACGTTTGCTTACCAAAATTCCCCAAAATTACTAAGGAAGTGGCAGATGGTATCAGA
GAAGGTAACTTACCAAATGAGAAGGACCCAAAATGCTATGTCAATTGTATCTTAGAAATGATGCAAACGATGAAAAAAGGCAAATTTTT
ATATGAAGGTTCCCTTAAACAAGTCGACATATTAATGCCTGATCATTTTAAAGATGAATATAGAGCAGGTTTAGCTAAATGCAAGAATG
CTGCTAATGGCATTAAGAATAGCTGTGATGCTTCCTATGCAATGTTCACTTGTCTTAGATCAGAAATAACTAGATTTGTATTTCCTTAATT
TGAAAGAGAATCGATATAATAATTTACACAATTTGAGTGTTTTTCAATGAAAAATTGGCCTGAAATTGTTTGGTTTGTAGAAAAAAAAAT
CGTGATGTAGTTTTAGATAA
>Cmeg29057_c0
GAAACATTCATAGATTTTGAAACCGGTATCACAACGATCTTCACCCTTAATGTCCTTGCACTTGTTCAAAACAGTCTTGGTCTTTTCTTCA
CCAATCAAAGCACCCAATTTTTGCAAAACTACATCTTCTTGTACTTGGCTGTCCTTCAAGGTACCAACCTTTTCAAAGAAGCAAGTACCG
AAACATTTCATGGCTGGTGAGGGGTTGGCAAAGTCCTTGTTGCGCAATCTGGTGGCATCTTCTTCAGAAACATTTTCTTGTTTAACACAT
TCTTGGAAGTGGACCTTAGCTTTGGCCTTTTGTTCTTCACTCAATTCAAGGGGATTGGCCAAAGCACAGGCAACGAAGCAAACAACAGC
TAAAGTGATAAAAGCTTTCATGTTGAAACTTTAGGTTTTTGTTTGATTAAGATGTTGTTTGGAAGACTGTTAAGAAAGTTGTGTTGTTCT
TTGCTTGATCATCGGGGGTTTTTATAACAAGAATAGTTTGCTTACATTTGACTTTTA
>Cmeg29840_c0
AAGAGCATTGCCAAGGAAAACAATTAAATAGAAGATTTTTTTTACTATGAAATTTTATATATTTTTAACGATTTTTATATTTTTTATAAAA
GAATCTTTGTGCAATTTAGAAAATGACAAAAATAGTGATATTATGCGTCAATGTTTGCAAGACAACAATCAGAATCCCGAAATTAGTAC
TGAAGAATTATTAGAGAAATTTAAAAATTATGCTAATTGGACTAATGAGGAAATACCCTGCTTTGCTCGCTGTGTGGTAGCGGAAAAAG
GTTGGTTTGATATTGAACAGCACAAATGGAATAAACAGCAGATTGTCGATGATTTGGGTGAAAATTTATACAACTATTGTCGTTATGAA
TTAAGTCGACCATTTCAAAATGTTTGTACTTATGCTTTCAAGGGTTTAAAATGTTTAAAAGATGCTGAGCTTAATGTGGTCGTGACCTAT
TCTCATCTCTTAGACTGTATCAATGAAAAGGCCACTAGTATGTCTCAACTTTTGGAATATTATCATTTTCCCAAAGGTGAACGTATACCCT
GTCTCTTCAATTGTTTTGCTGTAAGAGCCCAACTATATGATGATAATTATAATTGGATTATAAAGAATTGGCTTAAAGCATTTGGTCCTCC
AAGAGATTTAAATATGGCTAATGTTGCCGTCTGCCGTGTACCCGAAGAAAGAAGAAACCGTATGAATGTCTGCGCTTGGATGTATGAA
GAGTATAATTGTTGGGAACGTTTCAATTATAGTACAAATGGTTCGGTAGCTTATCGCAAGGCCTTAAAAAAGAGCAATGGCCACAAAAT
GTTTTAAACGTGGCGCTATAATTATTATTTTTTTTATAAAATTAAGTAAAAAAAATCTGAGAAAATTATTTTAAATTTTAAACAGTATTGG
CAGTAACAAACAGACAAAAATCACAATTTGACTGAATTTCATTGTAAGATAATTCATTTTTTATTTTAAGCAACAACAGCAATAAAATGC
ATAATTTAATATAATTATATTTTAGTCACACCACGTGTAATAACTAGGGTGAAGTTATTATTATTTTCTATTTTTTTTGGGTTTGCCAAAAA
CAAAAATTAATTTGACCCCCACATGAAATTGTATTATAATGTTTTGAAAACCTAAACTATTTTTTATTTATAAATTGAAATTAAAACTCTA
GTTTAAAATATGACTGGTCATTTTACATTATAAATAAACTGTTTGAGAAATATAACCGA

>Cmeg300820_c0
TACAACTAGTTTCCATTAGCAATACAACAATATCATAACACAAGCAATCGATATCCTCGAGATTAGATCAAGAAATAATGCACAGGATCT
GCTTTTTTCCAACACTGATGGAACCACCAAGCTTTGTGACACAAAGTATCACCTTCTGGATGTATACAATTTTCTGCCTTCATTAGTAGAT
CTCTGATAGAAATTGGTATATTTTTGAATAATGTTTCCAAATGTACATCACCATTATCGTCGACTACACCAAACTCATGGAACAAACAGTT
CATGTAACATTTCAAAGCTTCGTCTTCATGAATTTCTCCATCACTGAATTCCTTAATGGCCGCTTCAGTAACACCAGTCTTTCCTACACAAA
TATCATGGAATGGTATGGCCATTTTAAGAATTGCTGGTGGTGGCCAATCAGCATCACGCCTTGGTTGCTGTGCACGCGTCCATGTAGTC
ATCAACGACAGGACAATTAAAATTGTATAGAACAATTGAATACGGTAAAATGCTTTAAAAGCCATTTCTATTTCCGTCACAAGTGTAGG
CCTTTTAAAAATTTTTTGTTGTTAATTTTTTTCGAGTTCCTTTGCTACCAATTGTATGACAATTTTCGTTTAA
>Cmeg30479_c0

ACATTTTTCTTTTGTATTGATCTCTTATAAAAGCAATAAGAAGATACAAACAAATATTCAGTAACTTAACATACTTTCAGTGGAAGACAA
ATCTTATTATGAAATTTTTAATTTTGTGCATTTTCTACTTAGTGAACCACTCTATGGCAACGCCCCTTTATGAAGAGATTGAAATTGCCAA
AAAAGCTTGTCTAAAAGAACAACAATTAACCCGGGAAGAATTCTATAAAACTCCAAAATCTGAGCGAAAACTTGTATTGAAATGTGTGT
ATGAAAAAATTGGGTTTCATAAAGGATGGCATTATTCAAAATGATGTTATATTGGAAAAAACAAAAAAATTCTGATTTTGCAATACAAACC
AAATATGGTCTACGTTTATGCAGCAACATTAAACCTAATGATCCTAATGATTTGGCTTATGAACTAGAATATTGTTTTTCAGATATAAGT
GGAGAATATTTTGTAAAAAATTGGAAATTCATATGCGATAAATGCAATGAAGACATAAAACTTCCTAAAGATGAATATCAACTAATGCA
ATATCTTCCAGTTAAGAATATTACTGAATCTATGAAATTATATGCAAAATGTATATTGAAAAGTTTGGGTTTTCTAAAGGACAATAGTCT
GCAGGAACAGGATATTTTAGAAGTTATAGAAGACTTCAAAGATGCTAAATATTTGGAAGGATATTTGAAAGAATGTAAAGATATAAAC
GAACATAATGAAAATGATACAGCTTTTAAATTATATGAATGTATGGAAAATATTAAAGATAAATATGTTCAGAGCACAGAGGATGAATA
TAAATTGGCACAAAAAGATTGTCTAAAAGAAGAAGGATTCAGTCCAAATGAAGTCTACAATTCAGAATCACCTTATAAGCATATTTTTG
CCGATTGTGTTTTTGAAAAAATGGGTTACAAAAAGAATGACCTTATACAAACCAAAGTTATATTAGACAAATATGAAGCTGCAAAATTT
AGACAACATTACTTCGTTCTTCTTGAAAAATGCATCAACCCTACGGGAACCGATGATGACTATATTGATCATGATATAGAAGTTTATAAT
TATGCTCTATGTTTTGATCTATTTTCGGGTGAACATTTCTTAAAAGATTGGGAAAGAGCAGGTAATGAATGCAAAGATAGAATGGAATT
AATACACGATGATCAATATCCAGATATGAAAATGAAAAATCCAACTCCTGTTGTAAAATATTATGCTGCCTGTGTTTGGAAGAAACTTAA
TTATCTAAAGGCTAATATTTTGCAGGAAAATGTTATTTTGGAGGCAGCGGATATTTATAGAGATGCGGAATATTTAAAGGAATTTCTAA
GTAAATGCAATCGAATTAAAGAGAATGATCATATTGATACGGCTTATAAACTTTACCAGTGTATGGATAAAATTATAGAAGAACATAAA
AAGAAATAAAACTTTGTAATTTTGATTCCATAAAATCTAATAAAATTATGTTAATCCTGAAAATAATTTCAATATTAAATACAGGAGTACA
AGGATAAATCTATAGATAAGACTACATAATAAACAAGGTCCTGTGTAGGACAAATGGATGATGGTGCGCTGAAGACTATACTACGGAC
TAGACTGTAGAATATGCTATAAACTAGACTATAGACTAGACTATAAGTTAGTCTATAGACTAGACTATAAATTAGTCTATATACTAAATT
CGAAACTTAGCTATAGACTAGACTATAAACTA
>Cmeg31019_c2
CAGCACAGTCTACAAACGGAAACATTTCGAAACAAACAAAAATTTCTTAAAGTGAATAAATAGTAAAAATGAAAGCTTTTACAACTTTG
GCTATCTTCTGTTTAATTGCTGGAGCTTTTGCTCTTGAATTGACTGATGAACAAAAAGCCAAGGTCAAAGTTCATTTTGATGAATGTATT
AAACAAGAAAAGGTCTCCGAAGAAGAAGCTACCAAATTGCGCAACAAGGACTTTAATAATCCTACACCTGCTATGAAATGTTTTGGCAC
ATGTTTCTTTGAAAAAGTGGGTACCCTAAAGGATGGTGTTGTTCAAGAAGCTGTAGTATTGGAAAAATTGGCTCCTACTTATGGCGAGG
AAAAGGTTAAGGCTGCTTTGGCTAAATGTAAAGATGTTAAGGGCTCTGATCGTTGTGATACTGGTTTCA
>Cmeg32081_c4
TGCGATTTAAGTTCTAAAACTTAAACGTTTTGTCTCTTTTTTTATTAGCATTTATTCTTTAAAGCACATAATTATAGTATTAAGTTATAAAA
CTTTATAAATAAGTAGAGTTTGTTGACACTATAATTTAAAGGTCCCAGTATATATTTAATATTATTTAAGAAATGTCCATGTTATACAATA
GCAAATATGTTTTCAGCCATAGAGTCATTGAAATAAGTATAGGAAGCCAAGTAATCGTTGAGTATTTAAGAAATTAAATCTTCAGCAGA
TGAAATACCTTTACTTTCAAATTCTTGCTTTAAACAGTGACCATATTCTTCAGCAGCTTCACAGTGATCCTCACTGACTTCCAGACCTTCGC
AAGCCTCTATAACACCAGCTACTAGGGCTTTTTTGTCATCATCTTTGATCAAGGTTTTTGATAATTCTATGGCAGCATCTTTAATCATTTTA
CCTTCATCACTCATCACTCCAAATTTCTTTAAGGCACAAGCACGCATACATTTACCCTCCTTCGTATCGGCGGGTTGGTGTTTTATCATGG
CTTCAAAATCGTCATCAGAAGCTCCAGCTTCTTCTTTACAACCAGTTGCTATGGCTATTGCTTCTTCTTTTGTTAGATCAGCCCTTATATTA
CATGCAGCCAAAATTACAACTGATAATACCAAAAGGAACTTCATTTTTATTTTATTTTTGTAATTATATTTTATATATAATTTCTTTTAATA
ATTATATTTTTAATCAGTTTTATTTAATTTTAATGTAATTGAGTAATTTTCACTATTTTTATTTTTTAATTTAATTTATTTTTGACAATTTACC
CAAGTTTACCCGAAGAAAAAGTTCGTGCTGTTTTGTGTAATAATAACAGAATGTTATTCTGTTAAATTCTCTCTCATTTACCCAATTTTCG
AGTACGGGAACACTTTTCGGTAAAATTGGGATCCCTCGTGCGTCCAAAGTAGAGTAAGGAAACAGAG
>Cmeg32985_c1
TAATTTTAGTTATAAAACTTTATGTATAAAATAAAATACATAGTTCGTTGACACTATACATTTAAGTTCCCAGAAAATACTTAAACATTAT
TTAAATTTAAAAGAAATAATCATAAAAATCAAGAGAGGTCTAAGCCGTAGAATCATGGAAAATACATAGGATTTTTGTAATCGGTGAGT
ATTTAAGAAACTAAATCTTCAACAGATGAAATTCCTTTACTTTCAAGTTCACTGCGCCAGCAATGAGCGTATTCATCAGCAGCTTCACAA
TGATCATCACTGACTTCTAAAGCATCGCAAGCTTCAATAATTTCTACCATAAGCTCTTTCTTATCATCATCCTTAACAAGAGCTTTTGACAA
TTCTATAGCTGCATCTTTCACCATTTTGCCTTCATCACTTACTACTCCAAATTTCTTGAAGGTACAAGCACTCATACATTTACCTTCCATAG
TACTGGCTGGTTCATGTCTTACCATGGCCTCAAAGTCATCATCTGATGCCCCAGCTTCTTCTTTACAGGCCGTTGCTATTTCTAATGCTTCC
TCCTTTGTTAATTCGGCTCGTATATTACAGGCAGCCAAAATTACAAATGTCAACACCACAAAGAACTTCATTTCGTATAATTTTTTTGATT
GAAAACTTTTCGAAACTTTGCTATAACAAGTTTAAATGTTTAATGAACTAATATTTAAAAAGTTTCCT
>Cmeg33593_c0

TTTTTTTTTTTTTTTTTTTTTACTGGTCTTCTATTATAAAATTTTGATCTTTATTGTCAATTGATTTTTTGTTTTGGTTTAACATTTAATGTTTA
TGTACACTGTCCTTAACCAATTGGATATTGGATTGTATGAAACACATGCCACCACGATAGGCCCAAGTACAGGCATCACTACCTTGAGT
ATTCTTGTCGGCACATTTTTCGATTTTAGCATGAGTTTCATCATTATGATCAACGGCTCCATGTTCACCCACCAATTGATGATGAATCTTA
TGGACATCGAAACCTTTGTGTTCATCAAAGAAACCAAAATGTTCAAAGATACACTTCATATAACAATGAGTAGTTTCATCATCAGGATAT
TCCCAGTTTTTGTATTTCTCCATAAGTTCAGGGGATATTTTCAATTTATTGCCACAATCTTCACGATATTTCACTAAATCGGCATGATGTTT
AACCACATAATCATGACCATCTTGATGATCATGATGACCATGTTCATGATGAGCCAAGGCCGCTACGGCTAAGATTAAAAGCAAGAAAA
CTTTCATTGTTATTTATGATGGGTTCACAAATGCAACTGATCCCCGG
>Cmeg34233_c0
TTTTTATAAAAATCTAAAAATATATTTATGCATTTATAAGTAATTATTAAGAATAAGATAAAAATTCTATAAAAAATCTAAAATATTTAAA
AAAAGTATGTTTTTACTCAAAAAAGTAATATCACTAAGCTTGAGCAACACTTTGTTGCACCAACTGTAAATTGTTCTTAATGAAACAGGT
GGCACCACGATAAGCCCATTCGCAGGCATTTGAACCTTGTTCATTCTTATCAACACAGGAAGCAATCTTGGCATGGAGAACATCATCAT
GATTGGCTTCAGCATGACTGCCCAATAATTGTTGATGGATATTTTCAACATTAAAACCAGACGAAGTATCGAACAGACCAAATTTGGTG
AAAACACATTTCAAATAACATTGAGTGGTGGCATCATTGGGATATTGCCAGTTTTTATAGTGTTCTACTTGAGCTTCAGGAATTTCAAGT
TCAGCTACACATTCATTGCGATATTGCACAAGATTCTCACGAGTTTTCACCACATACTCAGCTGAGGTCAAAGCAAA
>Cmeg36113_c0
AAACTATAGCAGCAGAAACATTTTTATTGATAATAATTGCTGACAATCTAAAGATCATTATCGTTTATAACAATAACAACAATATATATTT
GTACTTGGAAAATCTAAAGCTCAAAATAGTTATAATCAAGTCTTATATATACAAACAACACAAAACGGCAGCCGGTAAAATAAAAATTA
ATTTTCTAAATAAATAAAACAATGAAATCAATAATTTTATTAATTATCAGCTTTAATCTTTTGGTTTTAATAGCTGCCCAAAGTAAAACTTC
TGATGAATTTCCTCAGAAATTTGAAAAATGTTTTCAACAATTACGTGTACCACAATCTTATAAGGCGAGATTTCAATCATTTCAATATCCC
AATGAAGAAATTGTACATAAATATATTCAATGTGTTTCCAACGAATTAGATATTTGGGATAATACTAATGGTTTTAATGTGGAAAAAATT
ACACAACAATATCGAGGCCGTGCCAATGATGAGGTAGTAGTACCGGTAATTTCTAAATGTAATCAAGATAATCAACATCGTAATAAGG
AATTGTGGTGTTATCGTGCATTTTTGTGTATCCTTAATACTCAAGTTGGCGAATGGTTTAAGGAGGATGTACGTCGTAAACAACAGAGT
AATATACCAAATGGTCATCATTAATGAATGGATTTTTTAATTAAAAAAAATATTAATCTTAATATTATATTTTATAGAGATTTTCAAATTG
AAATAAAATTTATTTAAATATGAATATAAATAAAATTATACAAGACCAAAATTCTACAAA
>Cmeg36536_c0
TCCAGTTGATTAGTCACATATTTTTTTCCGCTCAGCTTAGGATTTTGTTACACCTATAAAATGTTACTACAAACCTCGATTTTATATCAGTA
AATAGAACAATTTCAAACAGTTCAAATCATTTCTTTAAGCAATGAACAATATTACAGTTTTAGTAATTTGCTCAATATTAGCGGCCGCTTT
AGCAATGCCACCACTAGAACCACCCTCTTGGTATCCTGAAAATGCAGAGGAAATGGTAAAAAAATGTAACGAAGGAAATAACGTTAAT
CCCCAGAAAAAAATCGATATTTATACTGATACCCCCGAGTTGAGATCTCAGCTTTTGTGCAAATCTAAGGCATTTAATGTTTATACCGAG
GACGAAGGCTTTCATGTGGATCGCATGGCCTATATATTCTTTTATGATCCCGAGGGCAACAAAAGTGATCCCATTTTGCAGGATTGTGT
GAATAAAAATAAAAATATTTCTGCACATGACGAGAGAGTTTATAAAACTTTCAGATGTATTGCCGATATTGAAAAGAATGCTAAAAATA
AAGACTAAAGGATGTTATATACTTATAATGAAATAATAATGTTAATATAAAAAAAATGTCATTAAAAAAAAGTTAAAAG
>Cmeg614971_c0
TATAAAACAGAACACCAGTAAAATACAAGTGAAAATAAAAAATTATAACTTTTTACGTGTTATTTCCAGTAGTCCATAAAAAATCTAAAA
AAAGGAATAAAACAAAAAAATTCAAATCGAAATATGAAAAAATTGTTTTTAATTGAGGCTGCTTCTGTAGCACTAGTTGTCCTGATATTA
ATGCCGCTTTTAATTTCGGCACAGAAACCACGACGCGATGAAAATTATCCACCCCCAGATTTCTTAAAACGTTTCAGTATCATTCATGAT
GTTTGTGTGGAAAAGACAGGAGTTACAGAAGAAGCCATTAAAGAATTTAGTGACGGTGAAATACATGAAGATGCGGCTTTAAAATGTT
ATATGAATTGTATATTCCATGAAGTAAATGCTGTCGATGATGATGGTGAGGTGCATTATGAAAAATTGAAACGTTTAGTTCCGGACGAT
CTAAAGGAATTTGTTAGCCATATTATGGATGCCTGTGAATCTCATATACCCCAAGGTGGTACTCAGTGTGATAGGGCTTGGTCTTTTCAT
GTCTGCTTTAAAGAAACTGATCCTGTGCATTACTTTTTGCCTTGAAGTTATTATCTTTTTAAAACGTTTGGGTTATGGTA
>Cmeg8311_c0
GGACTCGGAATTTTTATTTTTTTGGAATTGAAAAGCACTATAGTAGTGCCTGCGTACCCTCAACTTAGATTTCTGGTCTTAATTGTGAACA
TATTTCCTACTAAATTCAAGAATTCTACAGAGATTGAATTATGGGAAATTTTAAGTTACTAATCAAACAAAATGTACCACTTTTTAATTAA
AAATCTTTTTTGGTTTAAATTATTAACTATAATTTATTTTAATCACATTTTTTCTACTTTTAATCATATATTAAACATCTTATTCTTGTTTTTG
GGCAGCAGCTTCATGACGTTTCTTAATATAGTTCTTAACTCTTTCACCGATTTTCGAGCTCATGAAACAGGCATGAGCTTCATAAGCAGC
AACATCATTGGGTTTATCACCCTTTGGATGTTTAGCAATACAGTCTTCAGCCAATTTTTGAACTTCTTCTTCTTCCATATCCAATTTGAATT
GCTTAGCAATACGATCGGCATGATAACCTTGATGAGAGCAGAAAATACCCATTTTCACAGCAGTGCAAAGAAGATATTGACGTACAGG
TTCTTCATTGGGGAATTCGAAATTTCTCATTTTAGTCATTTGTTCAGGGGTAAGAGGATGTTCCTTTAAGCATTCAGTGCGAATTTCTTTG
ATTTGTTCGCCATTCTTGACGGTCCACTCTTCAGCGGAGACACAAGCGATCAAGGCTAAAACTGCAATAAAAACTTTCATATTTGTAATT

TTATTTAAGTCGTTGTACAACCTTTTACCACAATGACTTTAACTTAAATGATTCAAATTTGTTTTCAGAAATGTATTATATACACATATATT
TTCTTCTACGACCGATTTTTAAGAAAACACACAATGAACACGTCTGTCCATTTTCCAGAACTGATAATACTTTTATATGTGTATTTGTATG
TTC
>Cmeg8717_c0
GAAACAGTATTTTATTATCTCTAGACCTCGTTGATTAACAATGAAATTTTTAGCTGTTATTACTTTTTTAACAATTGCTGCTGTTGCAGTCA
ATTCTCAAGCTCCGGCTCAAATACCTGAAGAACAAAAACTACGTATTATTGAATATGCTACAGCTTGTGCTGAAAAAAATTCCATTGACA
AGGAAGCTGTTCAGGCCCTTAAAAATGGACAATTTTCAAATGCCGATCAAAATACAAAATGTTTTACAAACTGCTTCTTAGAAAAGGCT
GGTCTCCTAATTAATGGACAAGTTCAAAATGATGTTATTTCTTCCAAATTGGGAAGCATTTTTGGTGCAGATAAAGTTAAAGCAGCTATG
GCCCAGTGTAATGGTTTAAAGGGTGCTGATAATTGTGAAACAGCCTTTGAATTGTATAAGTGTTACTTTAAAACAAATGCCGCTCTTATT
TAAAAGTATTAAGTAAAAATGTATTGTAATATGTTTTTTAATTTTTAGAAATAAAACTAAAATAAAAAGTATGCTTTAATCCGTAATTATA
GATTTTTGTGATTTAGTTGTTTATTGCGCTGATTATTTGTTAATATAATAATAACATTCGA

>Cmeg895_c0
GAGATCAAATATGAAAAATTTATTTTCTATAACGATTATTTCACTACTGGGCAATGTAATGACTTTGACCCTAGATCAAGAATTGAAAGC
AGCAGAAGATATATGTTTAAATGAAACACCCATATTTCCAGAACCCATACAATACAAACAATTTTACAATTGTTTCTTTGAAGAACTGGG
TTTCCTAAAGAACGGCACTATCCAAACCGATGTTATGTTGCAAAAGTCTAGCACTTTCAAATATCCCAACTTTGTTAAGAATTGTGTTCGT
AAATGTAAAGATATCAAAGGTACTGATCGTTATGATGTTGCAGTTAAACTTGGTCTCTGTATTAATAAAGTCAACATAAAATATTTTAAT
GACGAATGGATTATTATCAGCAATGAATGTAGAAAGGAACAAAAACTT
>Cmeg976228_c0
AAGACCAAAAGAAAGCCAATTGAGGTAGATACTATAGGAACTGTTAAATCCTTTTGTTATCTTAGTAGTCTCATAACAACCCCCGGAGG
GTCTGCAGAGGATATTATATACCGCATTAATTAAGACCGCTCCGCATTTTATTCCCTCACTAAAGTGTGGAGATCAACCCATATCTTCAG
AAACACCAAAATTCAAAGTATTTAATGCCTGCATTAAATCTG8
