# Supplementary material for: The developmental transcriptome of the synanthropic fly Chrysomya megacephala and insights into olfactory proteins
Source: BMC Genomics. 2015 Jan 23;16(1):20. doi: 10.1186/s12864-014-1200-y (PMC4311427; doi:10.1186/s12864-014-1200-y)
Supplement: Additional file 11: — S12-nucleotide sequences of CSPs. [file 12864_2014_1200_MOESM11_ESM.pdf]

>Cmeg21206_c0
TACAAACAAAAAATATATAATAATTTTGGCTTAAAGTATGAGGCCGGCCAGCTGATAGGTGTTCGATATAACGTATAAAAGGTATAGTG
GATTCACTACTGAAGTTAGTCGTTTGTCTACAGCTTTTAACTAAACAACAACTTGTTTTAGTTCAAAATCATTTAAACAAACTTTTTCAAA
AAAAAAATCAATAAAGAAAAAATGAAACTCGCCATTGTTGTTGTCGCTGCCTTCATGGCCATCGTCGCCGTTTCGGCTGATGAAAAATA
TACCACTAAATTCGATAACATCGATGTTGATGAAATCTTGAAATCAGATCGTTTATTCACCAACTATTACAAATGTTTAATTGATGAAGG
CAAATGTACTCCTGATGGTCGTGAATTGAAGAAGACTTTGCCCGATGCTTTGCAAACCGAATGCAGAAAATGCAGTGACAAGCAACGT
GAAAACACCGATAAGGTACTCCGTTTCATCATTGACAACAAACCCGAGGAATGGAAGGTCTTGCAAGCTAAATACGATCCTCAAAACAT
CTACTACAACAAATACAAGGATGAAGCTGCTGCCCGTGGCATTAAAATCTAAATCCTTTAACAACAAAAACTTAAAAAAAACATTGTGA
TCCAAAAAAATAACAATTTCTTTTAAAGTCAAAAAGTCGTCCTCAATACTCAGACCTAAACTGAACAAAAAAAAATCCCTTATATAAAAG
AAAAAAGGCTTTAAAATGAAAGAGAAAAAAACACAACTTTTTTGTACATAAAAAAATAACTTAATTGTAAATGTTTTGTATTATTTTGTT
AATTATAATCACGCGCTTTATGGACCCATTTAAAATTTCAAAATTATATTACATATTTTTTGTAAAATACCAAAAGGATGCACATTTTAGT
TTGTAGTTTTCTATTTGAAAAAATGAAAGAAAAATTCATTGGCTGTTAAAAATAATAAAATATTTTAAAATAATTATAAAAAAAAAAAAA
AAAAAAAA
>Cmeg25565_c0
GGTGGTGTGGTTTTATTTTATAATTTAGTTTTTCATAAAAAATATAAAAAATAATTCATAACAACTATTCCTCTTCCGTATGACCCTCAACA
ATATACTGTAAAACTTCTTCATCATTATGCATCAAGGAACTTATAACTTTATAAAAACCGGGACGTAGATACTTCTGAAATTTACTAGAAT
CCAAAGGATAATCTTTAACGACATAAATATCTTTTGTTATCGGACAATTATCAGGATCTGGAGCATTGGCATAATTTTTCAAAGTATCAT
AGAAATATTTTTTATACTGATTAGCCATAACATTACAAGCGCCCATATTAGCAAAACCAATAACTTTTTTATATTCTCCATCCTTTTCGGCT
GAATGAAAAACTTCCAAATTTAATTTATATGCATCATCTATATCGACGGACTGCTCAATTTGACCATTAATTTTCACCTCTTCTCCAATTTT
ATCGAAGGATATATCCGTTTTTAAAATATGCTCAGCATCATTGACTTTATCCAAGTTATCTAAAACCAATTTTAATTTTTTCTTTTCCTCAT
CGGCTGCTATAAATGAAACTGTTGTAATTATTAAATATCCAATAATAATTGAATATTTGTAATCCATGTTGAACCTTT
>Cmeg23554_c0
ATTGTTTAAACCTTTAAACATAACAGTTATCAAAAAAAAAAAATATCGAACACAAAATGAAGACAACTTTTGCCTGTTTATTGGTTGCGGT
TTGCTTCGCTTTAGTAGCCGCTCAAAAAACCTACACCAGCAAATTTGACAACGTTGATGTTGACAGCGTTTTGTCCAATAACCGTATCTT
AACCAATTACATCAAATGTTTGATGGACAAAGGAGCTTGCACACCTGAAGGCCGTGAATTGAAAAAACTTTTACCCGATGCCTTACAAA
GTGATTGCTCCAAATGTACCGATGTCCAAAAGAAGAACTCTCAAAAAGTAATCAACTTTTTGCGTGCCAACAGACCCGGTGAATGGAAA
TTGCTTTTGGACAAATACGATCCCAATGGTACCTACAGAGCCAAATACGAGAGACAAGGTTAAGCACGTGCAATGAATTTATATAGGGT
TCTTTTTCTTAAATACAAATTGAGATCTTTCAACAAAATTTACTTTTTTTTCTTCTCATAAATTATATAGTTTTTATACATTTTGGTATTATAA
GATAAAAAAAATAAAAACAAAAATATTTAAAAT
>Cmeg349412_c0
GTATACAATTTTTCTATAATTTCAAATTATAGCATTTAAATTTTGTGCACAAATACTAAAATATCAACACATATGAATAAATCGTCCTTTTC
AGTACCGGCCTTTGCTTGCATTTTATATTCTCCTTCGCCATTTCGTACCGGAAATGTCATATTCGTATGCTCATTAATATCGAAATCCAGTC
TTTGTCCTTCCGTAAATGGACAAGTCTTTTGGTCCTTAGGTGTATTTTCCATATAGGAATACCAATATTCGGTTTCACTAAAACAAGCTGC
ACAGGCATCATCTCGTTTTTGACTATAAACTGTGGGTTGCCAAGTACCTCGTTCTTTTTTAAATATTTCCACAGTAAACGGCATAGGTTTT
TCGACGGTAGTTGTTACAACACTATAGCCATTTACCAACATACTACCATCTTCAGTTTGATCAAAATTTACATTACTCATATTAAAAAGTT
GATCAATAAAATTATTCGGATATTGTTCACATGGCCTAAAGATTCTTTCATCCATAAATTCCAAAACGTATTCGTGTTGACAAGAAATCAT
CATTAACAAGCTTGTTAAAGTTAATAAAAATAATTTAAACTTCATTATTTTAACTTATCGGTTGCACTGATTAATTGTCATAGAATTTAATT
TGTTT
>Cmeg30884_c0
AAATCGATACCAAAAACTCCTTCTGCCTATTTTTCTGCTTAAATTAGAAGATTTTAATAGTATTAATATATTTATAATTAGTTTCATAGTGA
TTATTTCTTATTTCCGCTGGAGTTTTTAGATTTGATTTTATTTTTGCGTATTTGATTTGTGACTTATGGAAAAACATTTATTTTAAGAGTTTT
TTTGTATGATTGTGATTTAGTATTGAATATTTTAAATTTGCAAATAATTTACGATTTATTTTGCATTATTTTTTGTGCTTAATTTATAGGTAT
TTTAATTTATGTTTTATATTTTCTCAAAAGCATAGCCCAGACATCGGGATATTTCGTTTGTAAAAATGTTGTCAACTTCTGGGCATTTTGA
GCCTGCTGGGGTGAACAATTGCGACAGTTGCGTACAATAACCTCAGGGAGGGCAGCTTTAAGTTGTCGTCCCAATTGATCACAAGGAC
TCTTTTCCAAAACGCACATAATCTGACGACTGACAATAACTTGATTGTTTAGTAATTTATTTATATTCTTTTCATCACAGTTGACGACGAC
AGCAGCACAAATTAAATAAATCATAAACGACATAACAACTCGCGACATAATTGGTTTCATTTTCATTAAAAAAATGGGAAACAAATAAA
ACTTCCTTATTTCCTTCCTAGTTGCCTGACTAACTGACTGACTGTCTGATAGACAGACGGATTGGTTAATTGTTTAATTAATTGCCTTTAA
AATAATTTGCAGTGACGATTTCTATGTCTGTCTGAGTTATTTTTTATATGATGTTTTGTTGTTGTTCTTGTTTTAAAGGCTGACTAATTATT
TCTAGATGTTCTTGTTGTTGTTGCTTTTAAATCGTATCTTCTAGTTTGTATGTTTATGGAATTTTTAATCAACTTAATTTTATAAACAACGA

CACAACGGATGAAAATGATGACTGACATACACATACACACACACTCAAACTCACGTTATCGACTCAAAAATCACCGACTGACTCTATGA
TTTTTGAATATGTATTTTTGCCAATTTTAGAAATTTAAATGGGAAATACTTTTCACACAATGTTTTATTCTGCTGATGACTTCTGTTTATGT
TGACGAGTGGTACTGGAGCCAACCAGCCTGCT
>Cmeg23732_c1
AAAATTAAAAATTTTTGAAAAATGTTAAATATTAAAATAAATTGTTTATTGTTAATAATTTTATGTGTCTTAAGGGTTATACAAAGCGATG
GCGATGATGATGAGGACGAGGCGCCTTATACCGTGGAGATCGATAATTTTGAAATAAGCACGGAATATGATCACAAATTTATTAATTG
GGATACTTTGGGTTTAAAGAAAAAGAAACGTAATCAATTTGTTATAAGTGGCAATGTTATATTGAATCTAAATTTAGCAGATGATCAAA
AGATCAATCTTCAAATTTTCACTTATGATGCCGAGAAAAATGTTAAGGGTCCTTTGGTTTTTAATTTGGAAAAGGAGGTTTGTAAATTTA
TAGCTGAAGATGAGGATATGTATCCAGGAGTTAGAGCCAAATCCAATTTACCAGATCCAGGCACTTGTCCTGTACCTAAGGCTGAATAT
ACCATAGATGATTATATGGTTAAAGCCAATTTCTTGCCCGATGATATACCCAAAGGAGATTATCTTTTGGTGTTTCTATTAAAAGATGGC
CTGTCTCCAGTAGCTGGTTTTACGGCTGTCATTAATATAAGT
>Cmeg5343_c0
GCGGGTCTGCATCCAGATACTGATACAGATCCTAGAGAACTAGAAGCCTTTGCGGAGCGATTTAAGCAACGAAGAATAAAATTAGGAG
TTACACAAGCGGATGTTGGTAAAGCACTAGCGAATTTAAAGTTACCTGGAGTTGGGGCACTTTCTCAAAGTACAATATGTCGTTTTGAA
AGTTTAACGCTGTCGCACAATAATATGATTGCGCTGAAGCCCATCTTACAAGCGTGGCTTGAAGAGGCCGAAGCACAGGCGAAAAATA
AAAGACGAGATCCCGATGCCCCAAGTGTACTTCCCGCAGGCGAAAAGAAAAGGACTTCCATTGCGGC
>Cmeg22942_c0
TCCACTCTTAATAATTGAGCACAATGAAAAGGACATTATTGTCGTTGTTGTTAACGGTAATTAAACTCTGGGGACTAATTGAGTTTGGAG
ATTGTTTAGGCGAACAGGACTATGTAGTATCAAATGATGCTTTAATACCCATGGAAGGAGATCCGGAAACATTGATAGATTTTAGTCAT
TTAGAAACTGTGGGACGAAGACATTTAGTTAATGGTTCTTTGATCATTTTAGATGATATGGATGATGAAAATTTTGGTTTTACAGTGGA
AATGTATACTAGTCCTAATGGTGATGGTAATTATAAAAAAATGGCCATGGATGTTCCTAGGACTCAGGTCTGTGAGGGTTTTAAAAAAT
TCTATAAGGAATTTGTACAACCTAGCTTTACCTATGGTGAAAATAGCAATATTCCCTATATAGGAGAAGATGGTTTATGTCCTATACCTG
CCGGAGAGTACTATTTTAAGGATATAGAATTTAATACGGATGCCTGGCCTAATCAAATGCCTCGTGGTACTTTAAAGGTGGTGATAACA
TTTTATAATGGCGACAATATTGCTGGAGGTTTAACGGGTTTATTAAAAATTGAAAATGATACCAGATAAATTTTATAAAAAATAATTAAA
AACATTTTAAATTGGGATA
>Cmeg645582_c0
AATATCGAAATCCAGTCTTTGTCCCTCCTTAAAAGGACACGTCCTTTGATTATCAGGAATATCTTTTGCATAAGAATGCCAAAGTTCTGAC
TTACTGAAAATAGCCGTACAGGCGTCATTTCGTTTTTGACTGTAGATTGTAGGCTGCCAAGTACCACGTTCTTTTTTATAAATTTCTATAG
ATAACGGTAATATTTCTTCTACTGTACTTTTTAGAGTAGTGTAACCATTAACTACTATACTTCCATCATCGGTTTGTCCATAATCTAGAGTA
GTAACATCAAAAAGTTTATCCAAAAAATTATCCGGATATTGTTCA
>Cmeg386817_c0
CAAGAAAAAGGACTTCCATTGCGGCACCTGAAAAGCGATCTCTGGAAGCGTACTTTGCCGTACAGCCGAGGCCATCTGGGGAGAAGAT
AGCTGCCATTGCTGAGAAACTGGACCTTAAGAAGAATGTGGTGAGAGTCTGGTTTTGTAATCAGAGACAAAAACAAAAACGTATAGTT
AGTAGCGTTACACCCTCGATGACTGGTCATGGATCAACGGGTTTTGGTTACTGATAGTCGTTATTCCCTGGAGAGCACTACATGTAGAG
TTATCAACACCTCCCCAACACTAGGACTTGAGCACCCAACCAATATACACCAAGCGAAGATGACGATGACTGAGACGACAACGGTAAC
ACAGACGATCAACACGATGATGAAGAAGATGACTGAGAGGAGGAGCGAGGAAAAGACGATGCGAGAGCGTGAGTGCACTTATCCTA
ACACATCACCCCCATTCATCATGAAATGGATTTACACCCTATTTGCCAACAATCAAATGCGTATATTTATGGAACGATCAATGCAAACAA
TGGACCAACATATCAAATGCAATAACATTACTACTAATGATAAATTTTATATTGTG
>Cmeg425837_c0
TGTGTCCTGCTAAAGCAGGATTTGTTTTTAAATTTTTGATTAAAGTGCTTCAAAATTTATTCGCCAACGACTTTTGACGCCTCAAGGGCGT
TCAAATTGGAGGAAGATTTTGTGGGAGGCTGCATGACTATGTCGATGTATTCATCGACGGATAAAATGAAAATGTCAGCACCAAGTTG
TTTTCCAGGACGATATAGTCCCTCATATCGAAGTTCAGAGCAAATGAGGCGATGTATGCCAAATCCTTCGACTCGTTTGTTAGAAGATG
CATCCCTTTTATGCAATTCATGGTCAGCACGCCAGAATGGTGATATATTTGCTGGTATCAACGATGGCATACTCAGTAGAGCCGAAGCG
CTTGCGGCGGTTGACATTGGAAAACATCAAGCTTCTCACGTACATCCATCACAGATGACCTCCCAAATGAAACACGATGTAATGTATCA
TCATCACAGTATGGGCGGACCTCCTCAAAGACCATTGCAGATGCATCACTCAA
>Cmeg668690_c0
ACCATTGCAGATGCATCACTCAATGGACCAACTGGATATGTTGGATCCAACGGGTTCAATGACAACGCTCGCCCCCATTTCAGAAACGC
CTTTAACACCTACGCATCAACATTTACATGGATCCTACCATAGTATGAACCATATGATGAGTCATCATCATCCTAGTAGTTTAAGTGGAC
ATTCTACTGGCCATCATGGTCATTCTGCTGTACATCATCCTGTGATAACGGCCGCA
