# Supplementary material for: The developmental transcriptome of the synanthropic fly Chrysomya megacephala and insights into olfactory proteins
Source: BMC Genomics. 2015 Jan 23;16(1):20. doi: 10.1186/s12864-014-1200-y (PMC4311427; doi:10.1186/s12864-014-1200-y)
Supplement: Additional file 12: — S13- nucleotide sequences of IRs. [file 12864_2014_1200_MOESM12_ESM.pdf]

>comp3349_c0
GTACACTAACAACACCCAAGTCATTGTATCAAATTAGTCATTTAAATGAGATTTATCAAAATCGTATGCATATTATGGGTAGTGTAGACAATGT
GAGACATTTAAATAAGGAAGGAGAGATCTACCGTTATGTACGTGAACAGTTTCAAATGTGCTACAACATTGAAGAGTGCTTGCAACGTGCC
GCCAAAGATGCAAAACTAGCTGTAGCCGTCTCACGCCAACACTCTTTTTACA
>comp25409_c0
GGACTGCTGGCTTAGATTCTCCTTTAATGGTTAAAGATGAAGAGGCTATGAAAAATCTAACAGCCGATACAGTTTATAGGATTTTTACCGTA
GTGCAAGCCCCCTTTATAATGAGAGATGAAACAGCCCCTAAGGGTTACAAAGGTTATTGTATTGATCTTATCAATGAAATTGCCGAAATTGT
ACATTTTGATTATACCATACAAGAAGTG
>comp19139_c0
AACGAAGGAAGGAATAAAAAAGTGAATTAAATAAAAAATTTAAAAAATACGATTGATTTTAAAATGAGTTTAAGTTAGAGTACGAATAAAA
AATAACGTTAGGAAAAAAGGGGCAAAAAAATATACACAATCTACTGAATATTTAACAGAAGGAGGTGGAGGACGATAAGGGGAGTCGAA
AGTGAAGAGTTGGGTTTTTTTTGTATTAAAACCAAAGTTTAAATTTAGAATTAAAATTCCATGAGATAGGGAACGAGTATGAGTGAGTGAG
CAAGAGGATTCAAGACGAGTTTTTTTTTTT
>comp3349_c1
GCCAACACTCTTTTTACAATCCTCGCATACCACGCGATAGTTTATATTGCTTTGATCGCAATGAAAATATCTATGTTTATTTGGTGACAATGTT
GATGCCAAAGAAATTTCATTTGTTGCACAAAATCAATCCGGTCATACAACATATAATTGAATCGGGTCACATGCAAAAATGGGCACGAGATT
TGGATATGAAACGCAAAATAAACGAAGAAATACAACGTGCCCGAGAGGAACCATTCAAAAGTTTAACACTATCACAAGTGGGGGGTAGTT
TTGCATTCAATGGTATTTTGTTGCTATTTGCCTCTTGGATGTTT
>comp25409_c3
GCTAATATTGAACAAATGTTTTATGAAATTTGGAAAGATTTATCTTTAAATGATTCTTTAACGCCTTTGGAAAGATCTAAATTGGCTGTTTGG
GATTATCCTGTCAGTGATAAATACACTAAAATGTGGCAAGCCATGCAAGAGGCACAATTACCCTCTACACTAGAAGAGGCAGTAGCTAGAG
TTCGTAATTCTACTACGGCTACAGGATTTGCTTTTCTGGGAGATGCTACCGATATACGTTACTTGGTAATGACCAATTGTGATTTGCAAGTAG
TGGGTGAGGAATTTTCACGTAAACCTTATGCTATAGCTGTACAACAGGGTTCACATCTTAAAGATCAATTTAATAACGCTATATTAACTCTACT
CAACAAACGACAGCTGGAAAAACTCAAAGAGAAATGGTGGAAAAATGATGAAGCCCAAGCTAAATGTGATAAACCCGAAGATCAATCCG
ATGGTATTTCAATCGAAAATATTGGTGGTGTATTCATCGTAATCTTTGTGGGCATTGGCATGGCCTGTATTACTTTGGTATTTGAATACTG
>comp20304_c0
GGCACCAAATATAACACTTATTAGACATAAGGAAGCAATAAAGACACGTTCTGAATTAAAGGGTGGGTAATGTATAATATTTACTCTGACCC
ACACTACCCAAGTATCAATAAGTATACGTACGTATTGCCATCTAAAAGTTTTCTGCAAACGACTTCCGCGTATTTGAAATTTATTAAGTTGCAA
ATTGATTATACGCAAGAAAGCCCAAAAAAATGAACAGAAAAATGCCATTAATATAAAACCAAGCCATAGATCATAACGTACAGCAAATATAG
GCAAAATAGATTCCGGAATTTTTTTAGCTTTCGGTGTATAAATACATAGTTGATCGTCATAAACTGCAACGGAAAAATCCATTTCTGGTACCA
TGTAATCTTTAACAAAAAATCCGGTTACACATAAATCCAATTCGCCTCTTACTATAGATCCAATAACACCATTATATGAACCATTTGGACTACGT
TCTCCAAAATAATTTGGATCTGGATCTT
>comp1881_c0
TTTTTATAATTTTGGGTATAGCGGTTAAAGATAAAATCGTACGGGCACAAACGAGTCAAAATATTAATGTTTTATTTATAAACGAACTTGATA
ACGATCCAGCCACCAAAGCTATTGAAACGGTACAAACGTATTTGAAGAAAAATTCAAACTATGGATTGTCGTTGCAAATCGATAAAATCGA
AGCAAATAAAACGGATGCTAAAGCACTTCTGGAAACGATTTGCACCAAATATGCCCAAAGTATTGAAAATAAACAACCGCCACATGTTGTA
TTCGATACTACAAAATCAGGAATAGCTTCAGAAACGGTTAAATCTTTTACACAAGCTTTAGGTCTACCCACTGTAAGTGCCTCTTACGGTCA
AGAAGGTGATCTTAGACAATGGCGTGACATGGAAGAAAGTAAACAAAAATATTTATTACAAGTTATGCCTCCAGCTGATATAATACCGGAG
GTAGTAAGAAGTATAGTGCGTAAAATGAATATAACCAATGCCGCCATTTTGTATGATGATACTTTTGTTATGGATCACAAATATAAATCTTTGC
TGCAGAATATTCAAACACGTCATGTAATAACGGCAGTGGCAGATGGTGAACGTGCCAGAGCTGATCAGATAGA
>comp12593_c0
TTTGATAGCGTGAGGAAAGCTGTTAAATTTGCAGTATAAAATGAGGTCAATATTGTGATGAATATCCACCAGGTTGCAAATAATAAACGTGT
TGAATCTGCAATTGGCGATAGAATACTGCCTTGTTTCATTAAAGCACCATAAACAAACCAGGCACAATGCCCCAACGAATAAGGTTTCTGCT
CCGTATCGCCAGTTAATTTATTGCGCAAAATTATCAAAAAGTAAATGATGGGACCAACCGTCAGCAAAGAAATAAAAATCAAAATCCAAACC
CAATAATCAAAAGGTGCCAAGAGGCCAGAACCTGAAGCCGATTCACTAGGACGTTGCATAACCATAATCCATTCGCCTTCATCCAAAGTGG
TGGTTGAGTAAAAAACAAATGTGCGCTGTTCAGAGAGTAAGGGCAAAAAGGCAGCTGCCATATCCGCTTCCGTGGTATTTAAAATCTCTAT
TAAACTGTTGGCAAAATCTGTTTTCGAACCAATTATGTTTCCCTCCGGTACCACCACTTCATAGGTAAAGTTGAATTTCTCCTTTAAGAAATC
GATCAATTCAAATGCCACACCCAAACCCACAATTGTACCATTTTCATAGTGTTTGGTATAACTTAAAGGATAGTCCTCCAAAGTAGCTATACG
CAAGTGTCGA

>comp22717_c0
AAAAAGTGTCAAGTTTTAAAGCCCACAAATAAATTTAAAGAAAAATCTTTTACTCACCGCTGCATATTTCTCAAAATATGTTCCATTACGCAA
ACCAAACTGTTCATATTCGTTATGTTTGAAAAGTTGAAAAAAATAATCCAAACCCGGTTGAAATTTGGGAAACGTTAAAAAAGCCACCAGA
TTACCACAATATGTAGTCACCAAAACTATAACAACAATCCACCAGAAACCAATAACCAAACGACCACTATCAGCTTGAG
>comp475644_c0
TCGTTATCGTAAAACTATTAGTATGACGAATGAGAAGATACATTGTCCCTTTGCTAAGGCTAAGGAACCGTTTTTGAAGAAGAAACGTTCAT
TTGCATATCCGATTGGCAGTAATTTGAGTGAATTATTTGATAGAGAATTACTAAATCTCGTTGAATCGGGCATTATAAAACATCTTTCGGCTAA
AGATCTACCAAATGCCGAAATATGTCCACAAGATTTGGGTGGTACTGAACGACAACTGCGTAACGGTGACCTTATGATGACCTATTACATTA
TGTTTGCTGGTTTCGCTACAGCTATAGTAGTATTTAGCACTGAACTACTATTTCGCTATATCAATAGTCGTAACGAAAACAATAATCAATGGGC
AACACATGGTGTGGGTCGTACACCGAATGGTCATCAATTTAAGCCCTCGAAATGGTTTTGGCGTAAAAGTGGTACGGATAGTGCTAAGAAA
TTGCTTAATAGCTCTCAGCATAGTAATATAACACCACCGCCACCATATCAAAGTATATTCAGTAGTAAGGGACGTCAAGATAATAGCAGTATG
AAGAGATGGCATCATGCAGCAAATTTCGGAGCTAATGGAAATGGTGGTTTTGGTGCTTTAAGGCCGGCTGTGGCGGATTCTCAGTACGGT
GGTATGGGTATAACATCGACTGGTTTAAGGAAATTTATAAATGGTCGTGAATATATGGTATATCGTACACCAGATGGTCATAGTCAATTGGTA
CCGGTTAGAGTACCCTCGGCAGCTTTGTTTCAATATACCTATACCGAATAAGGGG
>comp25409_c2
TGATTATACCATACAAGAAGTGGAAGATGGTAAATTCGGTAATATGGACGAGAAGGGAGAATGGAATGGTATTGTCAAGAAATTAATTGAC
AAACATGCCGACATTGGTTTGGGTTCAATGTCTGTAATGGCAGAACGTGAAATTGTTATAGATTTTACTGTGCCCTATTATGATTTAGTGGGT
ATTACCATAATGATGCAACGTCCTAGCACTCCCAGTTCTTTGTTTAAATTCTTAACGGTTTTGGAAACTAACGTATGGTTATGTATATTGGCAG
CCTATTTCTTTACCAGTTTTCTAATGTGGGTATTTGATCGTTGGAGTCCTTATAGTTATCAAAATAATCGCGAGAAATATAAGGATGACGATGA
AAAAAGAGAATTTAATTTAAAGGAATGTTTATGGTTCTGTATGACTTCTTTGACACCTCAGGGTGGTGGAGAGGCTCCAAAAAATCTTTCG
GGTCGTTTGGTAGCAGCTACTTGGTGGTTATTTGGTTTCATTATCATTGCTTCG
