# Supplementary material for: The developmental transcriptome of the synanthropic fly Chrysomya megacephala and insights into olfactory proteins
Source: BMC Genomics. 2015 Jan 23;16(1):20. doi: 10.1186/s12864-014-1200-y (PMC4311427; doi:10.1186/s12864-014-1200-y)
Supplement: Additional file 14: — S14-deduced amino acid sequences of the identified OBPs. [file 12864_2014_1200_MOESM14_ESM.pdf]

>Cmeg12669_c0
MNNLIYLICSLLGTTIAAPPSWYPNNAKELTKKCMEDNNITPEMAKRYGSLYDNPIRRTIMLCNVKSSNIYSEETG
FYVDRMAYMFYEPASYVGARKAILQNCVDEHKDLSSHAEIAFNTVKCVLDTDDKEISNLNKN
>Cmeg16201_c0
MGKLFVVFTILGIFAAVSVKGFDKETAKAILREKAEMCKEKVGASESDITNIIDQKPSSTKEGKCLRHCIMEHFKV
MDENGNFDKDIAMTHVRFLTDGDEDKINTASEIIDHCTGTVAEDDKCEVAEQYDICFMEQAKSHNIDDIFEM
>Cmeg20107_c0
TCFFEKIGVLKNSVVQESVVLEKLSSIFGEEKIKAFLKKCKNVKGSERCDTGFKLYQCFEKA
>Cmeg21243_c0
MKVYILLITVILAVAQVKCDLKEDIRQANMACLEETKANQDELKSFFKGELKGEPKEALKCHLKCFMEKQGQWK
NGAFDEKSAIKYLQNIPALKDHQDAINKAMNDCKTQKGSNECDTAYLIMKCLGEHKASMM
>Cmeg21269_c0
MKAFITLAVVCLIAGAFAHLELSDEQKAKVKVHFDECVKEEKVSEAEATKLRNKDFANPTPAMKCFGTCFFEKLG
TLKDGVVQEAVVLEKLAPHFGEEKVKTVLDKCKDIKGADRCDTGFKIYECFEKAKAELGH
>Cmeg21549_c0
MGKLFVALAILCVFGSVLVQGFDKEAAKAALKEKAQICKGEVGASDSDIAELTEKKPASTKEGKCLRSCIMKKCKV
MNDNGKFDKDTAMSHAKMYTDGDEDKMKIATEIIDHCSVIDVDDDECEAAEQYG
>Cmeg21654_c0
MKRVLIVLFIICFFQYISATSIANEFRSADFECKKELKISKEDASKLLQSIENPTLETKLYFNCFLEKLGFLKDNILQENY
ILSKTDEYSDAGYLRKFVSKCKHIKGEDQYETAVQLGICFHNTNRKEEF
>Cmeg22689_c0
MFKFTYNLIIFCLIGIVIVKALEEGDMKAMVLKVGEGCKAETGASDSDLQELLDSKFASTKEGKCLRFCVMKKIG
QIDDNGKLRKLPYTEYMTEKIKTGVEIINQCTDIEVDNDPCVAIEQYEQ
>Cmeg23484_c0
MKATLAILIALFALVSAEYKLRTQEDLVKARKECMEAKKVSAELIEKYKKFEFPDDEVTRCYIECIFEKFELFDAKDG
FKNDNLVAQLGHGKEKKDEVKADVEKCADKNEQKSDSCAWAFRGFKCFISKNLPLVMESLKKN
>Cmeg23600_c0
MLKSVISLLLIATLQSTLATIIDCQRPPQLVDPAKCCLDGGRDEVTEYCAQKMGITGRPSDAPPTVETATCLAECILT
ESKYMQKPETLEIGIIKSDLYNKFSNDTIYAETMAEAFAKCQPTAQRKMKAFKQIPLGNVALQRGCSPFAGMVLG
CTYMEYFKNCPAHRWTENAECSLAKQFVTQCSLGA
>Cmeg23854_c0
MKLIITLAIFCLIAQACTSPLELNDEQKAKAKEYFQECIQQEHVTEEEAAKLDKKDFSNPSMNLKCFGTCFFEKVG
TLKDSVVQEDVVLAKLGSILGEEKTKAILEKCKDVKGEDRCD
>Cmeg24434_c0
MKLQVALILVMLAVVSAKFEIRTSEDAIRNHEECREEYNVPDDVYEQFLDYKFPEHKNTKCYIKCWVEKMGIFNP
KKGYDEKAIIAQFTHGNPKFLSSVQHGLEKCIDHNESESDVCTFAHRVFTCWIKSNRHAVRKILGTN
>Cmeg248939_c0
MGHLQKLILFCLFTFSWSANPSLNRYEAARKFQLILEECREEVAATTADIQELLNRKPASTMSGKCFRSCLMKKY
NVMDSDGKFDKEAALDEARKLTRGDVSKMQLAENLTNACSDIEVSSDHCEAAADYGECFREQIKSLNLAKS
>Cmeg24919_c0
MKIFITLALFCLLGNSLSESLDEEFKAAEDDCLKEKHLTRNEVFGDNDLTNPKPGLKMFAGCFLGKVGFIKDNVM
QEDIMLKRSKDFKYPNFFKHCVRVCRHTKGIDHDDTAYKMGLCFRKVDTSDFLKEWKLASKDCKEEQQLSEEEY
TKLQHLPVDNPTPAMKQYTACVFEKEGFLKDNILQENVILEVSEDYKDAEYLKTFVNECKHIKENNQTETAFKLFE
CLQKIKDTHIELLKKE

>Cmeg24940_c0
MMRIFILLVVICLIANVFAVNHAQLVEILKAKNRIPFDVCLIQERVSEEEGTELLNNVTTKPRPAVKCFGSCFFEKIG
TWKDGVIQEDLVLEKVGPIFGKERVENAIKTCRHIKDSDRCETMYKFIKCYIEA
>Cmeg24941_c0
MNTVFLSHLLLVAVLLLSFKQHTDAAPNADAAKKAAEECIKEGGLNAEDSKRIMANELFSPKYETASDKLQCFLL
CYYKKIGIIDAIGKQKADVFMGYLEHRFSDKKDKIKPALAKCSTVKATNPCEAVYAFEACVLKNIN
>Cmeg25217_c0
FGTCFFEKIGTLKDGVVLEAAVLEKFAPAYGEEKVNAALTKCRGVKGSDRCDNGFKILECFEKAKDEVGIV
>Cmeg25756_c0
MKVFIVLGLIFLLGNAKAATLQDELNAAEDTCIKDNKLNRDEVFGDYTKPTDSLKIFAGCLLGKVGFVKNGVVQK
EVILEKTKLTKYPKYFKHAARICSHITGTDHNDVAYNLGMCFKKVNGEIFLLEWKNSGMECKEEEQLSDEDYTKL
QNLPIENPTLAMKRYTHCILTKQSFLKDNVLQESVILEVAEDYEDADFLKTFVNQCKQIKEDDQNDTAIKLYVCML
KNNKA
>Cmeg26369_c1
MKAFTTLAFLCLMAGAFAQFQLTEEQIVKAKAHFDQCVKQEKVSEQEATKLLNKDFVNPSPAMKCFGTCF
>Cmeg26380_c0
MKIAHEIIDACVGITVPDDHCEAAEEYGKCFKKEYLAHGLKEIEIDF
>Cmeg26380_c1
MAKIFVTLTILCVFGAVLVKGFDKKKAIADFMTRIDECKVEVGAKDTDVEELLEKKSASSMEGKCLRSCLMKKYE
MMDSNGKIVKDKVVTDAEKYTDGDADKMKIAHE
>Cmeg26644_c0
MSKFLLNLLIFCLIGIVVVKGLSEEETKTIIKSIATGCKPESGAKDSDIEDVLDNKFARTKEGKCLRFCVMKKMGQM
DDKGKLKTLPILKSLSENTKTGGEIMNQCVDIDVDNDPCEAMDQYEHCFNEKATAVNFKDLMYT
>Cmeg26709_c0
MAKILLTLTIVCVFGAVLVKGFDKEKAIAGFMAKMDDCKAEVGAKDTDVEELVGKKPASTMEGKCLRSCLMKRY
EVMDGNGKFVKDVAISHAEKYTDGDEDKMKIAHEIIDACSGLDVSDDHCEAAEQYGKCFKDESIAHGLKEKLN
F
>Cmeg27265_c0
MNLLPKLLICFSIIVLTYADDDGMSVKDIAEALMSFGEDCEPKAEEEHIIEVVKNVKDAQYTSKCFRHCLMSQFEL
IAEGSTTMDKEKTVDMMGSMFSDRKDDLSQIIDECNTKNEAITEKCENAHAHGMCMLDLMKERGFDIPDLK
DE
>Cmeg27557_c0
MTTLESLINRSKMKTFNTIFLNLCLVTLVVGNCDKNLDPKEGKAIAECLDRFGGPTLENAQRLQRFRQWSDTYE
EIPCFTNCYLNKMFDFYNETTGFHGPNVIKHFGNVIYEACKNKLIEGNDACEIAYNGFHCMVNLENDPFVVIES
MQGLNADAKLAMKDCLHKFDQFEWQKFGEYSSYPVKEPIPCYTRCFVEKLQLFNHRLRKWNTANMLEKLGFP
EENANISNCLAMGQRRSRNSCAWMYREFICFLMSRGVKNVQNKL
>Cmeg27729_c0
MKSFITLAVLCLIAGAFAQNNKFARTTELLKLHSRAHIDVCIKQENISEEDVTKLFSNELTDPSRNIKCFGACYVERV
GTWTNGTLQEKVILEKLVPIFGEEKIRTALTKCNNVKGSDRCDTVFKIIKCFLEA
>Cmeg28108_c1
MKIFIVLFIFYLLDYSLAVSFYEENEIAENDCLREQQLTAEDFYRISRAQRTILFKCIYEKLGYIKDGIIQTNVILEKSKN
SAIANKYKYGVRLCEYIISNDEHDLAYKLSECFKKTNPDYFQRNWNILDYECKDKVKISKYDYKRLQYFPLENITEP
MKLYTKCMMKTLGFLKYNNLQEKVVLEVSEDFEDAKYLKAFLKECKGIKEYNENDTSFKLYECLENVKEKYVLSTE
DQYKLAQKSCLQEKQLSPHEVYHQQSPYKQMFANCVFEKMG

>Cmeg28108_c0
MKMKNPTPAVKYYAACIFKKLGFLKANTLQKNVVFKALEDYRDAEYLKIFLSKCKHIKEKEHIDTAYKLYQCMDKII
HTEKKDDRE
>Cmeg28341_c0
MAYGFLVLTILAALGHTCLAAGVDCKKMPPKVDPASCCPIPELITEENKEACKEFLMEPKPSFVPMGNEKPIGTA
NKAAPTPASHRSDHYHRIRNEPLMHLCFMNCALNDTGILTNAKLNSAALTTQLKKVLNDTPDLIPVLETSFKTCS
AMGEKFHQKMQERMKKRKMSTTPATATKDRMLRPLRCPPIASHMMACVFMETFMKCPASVWTKTNECNE
LRDHMLNCQPKYSMEESSEEDAM
>Cmeg28677_c0
MAATKLKLFRLVLIVAGFAATKAEVDCKKHHPHVDMKSCCEISNFVSDEIKEKCEVEGPPGPPGVGTPGDVPPG
PPGGHGPHGHHHGGPHGPHPCFVACALNETGILIDDELQEDNLQTYLNGVFDDGDKVEFLMEKFKYCDEKRK
NSGEKQHHGHGPPHHHHHNCGGPKHGKMLVGCVFMETFKECPDNAWSNTDECNEARDHFIQCPHFGPGE
KPQEEDVEAI
>Cmeg28701_c0
MILKQNLIFLIGIVVCICKITEVFGGATEEQMWAAGNLMRDVCLPKFPKITKEVADGIREGNLPNEKDPKCYVNC
ILEMMQTMKKGKFLYEGSLKQVDILMPDHFKDEYRAGLAKCKNAANGIKNSCDASYAMFTCLRSEITRFVFP
>Cmeg29057_c0
MKAFITLAVVCFVACALANPLELSEEQKAKAKVHFQECVKQENVSEEDATRLRNKDFANPSPAMKCFGTCFFEK
VGTLKDSQVQEDVVLQKLGALIGEEKTKTVLNKCKDIKGEDRCDTGFKIYECF
>Cmeg29840_c0
MKFYIFLTIFIFFIKESLCNLENDKNSDIMRQCLQDNNQNPEISTEELLEKFKNYANWTNEEIPCFARCVVAEKGW
FDIEQHKWNKQQIVDDLGENLYNYCRYELSRPFQNVCTYAFKGLKCLKDAELNVVVTYSHLLDCINEKATSMSQ
LLEYYHFPKGERIPCLFNCFAVRAQLYDDNYNWIIKNWLKAFGPPRDLNMANVAVCRVPEERRNRMNVCAW
MYEEYNCWERFNYSTNGSVAYRKALKKSNGHKMF
>Cmeg300820_c0
MAFKAFYRIQLFYTILIVLSLMTTWTRAQQPRRDADWPPPAILKMAIPFHDICVGKTGVTEAAIKEFSDGEIHED
EALKCYMNCLFHEFGVVDDNGDVHLETLFKNIPISIRDLLMKAENCIHPEGDTLCHKAWWFHQCWKKADPVH
YFLI
>Cmeg30479_c0
MKFLILCIFYLVNHSMATPLYEEIEIAKKACLKEQQLTREEFYKTPKSERKLVLKCVYEKLGFIKDGIIQNDVILEKTKN
SDFAIQTKYGLRLCSNIKPNDPNDLAYELEYCFSDISGEYFVKNWKFICDKCNEDIKLPKDEYQLMQYLPVKNITES
MKLYAKCILKSLGFLKDNSLQEQDILEVIEDFKDAKYLEGYLKECKDINEHNENDTAFKLYECMENIKDKYVQSTED
EYKLAQKDCLKEEGFSPNEVYNSESPYKHIFADCVFEKMGYKKNDLIQTKVILDKYEAAKFRQHYFVLLEKCINPT
GTDDDYIDHDIEVYNYALCFDLFSGEHFLKDWERAGNECKDRMELIHDDQYPDMKMKNPTPVVKYYAACVW
KKLNYLKANILQENVILEAADIYRDAEYLKEFLSKCNRIKENDHIDTAYKLYQCMDKIIEEHKKK
>Cmeg31019_c2
MKAFTTLAIFCLIAGAFALELTDEQKAKVKVHFDECIKQEKVSEEEATKLRNKDFNNPTPAMKCFGTCFFEKVGTL
KDGVVQEAVVLEKLAPTYGEEKVKAALAKCKDVKGSDRCDTGF
>Cmeg32081_c4
MKFLLVLSVVILAACNIRADLTKEEAIAIATGCKEEAGASDDDFEAMIKHQPADTKEGKCMRACALKKFGVMSD
EGKMIKDAAIELSKTLIKDDDKKALVAGVIEACEGLEVSEDHCEAAEEYGHCLKQEFESKGISSAEDLIS
>Cmeg32985_c1
MKFFVVLTFVILAACNIRAELTKEEALEIATACKEEAGASDDDFEAMVRHEPASTMEGKCMSACTFKKFGVVSDE
GKMVKDAAIELSKALVKDDDKKELMVEIIEACDALEVSDDHCEAADEYAHCWRSELESKGISSVEDLVS

>Cmeg33593_c0
MKVFLLLILAVAALAHHEHGHHDHQDGHDYVVKHHADLVKYREDCGNKLKISPELMEKYKNWEYPDDETTHC
YMKCIFEHFGFFDEHKGFDVHKIHHQLVGEHGAVDHNDETHAKIEKCADKNTQGSDACTWAYRGGMCFIQS
NIQLVKDSVHKH
>Cmeg34233_c0
FALTSAEYVVKTRENLVQYRNECVAELEIPEAQVEHYKNWQYPNDATTQCYLKCVFTKFGLFDTSSGFNVENIHQ
QLLGSHAEANHDDVLHAKIASCVDKNEQGSNACEWAYRGATCFIKNNLQLVQQSVAQA
>Cmeg36113_c0
MKSIILLIISFNLLVLIAAQSKTSDEFPQKFEKCFQQLRVPQSYKARFQSFQYPNEEIVHKYIQCVSNELDIWDNTN
GFNVEKITQQYRGRANDEVVVPVISKCNQDNQHRNKELWCYRAFLCILNTQVGEWFKEDVRRKQQSNIPNG
HH
>Cmeg36536_c0
MNNITVLVICSILAAALAMPPLEPPSWYPENAEEMVKKCNEGNNVNPQKKIDIYTDTPELRSQLLCKSKAFNVY
TEDEGFHVDRMAYIFFYDPEGNKSDPILQDCVNKNKNISAHDERVYKTFRCIADIEKNAKNKD
>Cmeg614971_c0
MKKLFLIEAASVALVVLILMPLLISAQKPRRDENYPPPDFLKRFSIIHDVCVEKTGVTEEAIKEFSDGEIHEDAALKC
YMNCIFHEVNAVDDDGEVHYEKLKRLVPDDLKEFVSHIMDACESHIPQGGTQCDRAWSFHVCFKETDPVHYF
LP
>Cmeg8311_c0
MKVFIAVLALIACVSAEEWTVKNGEQIKEIRTECLKEHPLTPEQMTKMRNFEFPNEEPVRQYLLCTAVKMGIFCS
HQGYHADRIAKQFKLDMEEEEVQKLAEDCIAKHPKGDKPNDVAAYEAHACFMSSKIGERVKNYIKKRHEAAAQ
KQE
>Cmeg8717_c0
MKFLAVITFLTIAAVAVNSQAPAQIPEEQKLRIIEYATACAEKNSIDKEAVQALKNGQFSNADQNTKCFTNCFLEK
AGLLINGQVQNDVISSKLGSIFGADKVKAAMAQCNGLKGADNCETAFELYKCYFKTNAALI
>Cmeg895_c0
MKNLFSITIISLLGNVMTLTLDQELKAAEDICLNETPIFPEPIQYKQFYNCFFEELGFLKNGTIQTDVMLQKSSTFKY
PNFVKNCVRKCKDIKGTDRYDVAVKLGLCINKVNIKYFNDEWIIISNECRKEQKL
>Cmeg976228_c0
KTKRKPIEVDTIGTVKSFCYLSSLITTPGGSAEDIIYRIN
