# Supplementary material for: The developmental transcriptome of the synanthropic fly Chrysomya megacephala and insights into olfactory proteins
Source: BMC Genomics. 2015 Jan 23;16(1):20. doi: 10.1186/s12864-014-1200-y (PMC4311427; doi:10.1186/s12864-014-1200-y)
Supplement: Additional file 15: — S15-Phylogenetic analysis of OBPs of Chrysomya megacephala and Drosophila melanogaster. [file 12864_2014_1200_MOESM15_ESM.pdf]

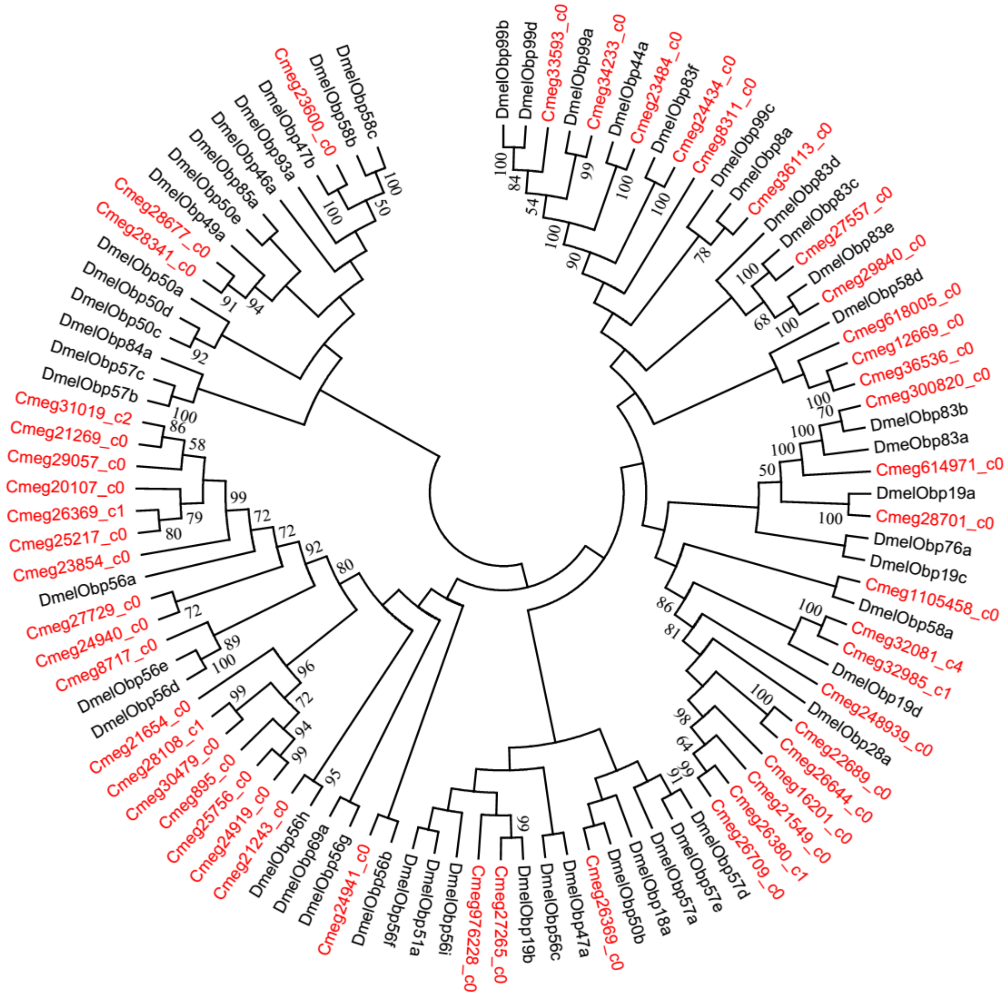

Phylogenetic analysis

Fifty-one Amino acid sequences of *Drosophila melanogaster* odorant banding proteins (OBPs) were derived from Hekmat-Scafe, et al. [1]. Forty-seven *C.megacephala* OBPs were used for phylogenetic analysis. All the senquences of OBPs were firstly aligned with clustalX2 [2]. Then unrooted trees were constructed by MEGA6.0 [3] using Neighbor-joining method with p-distance model [4,5] based on 1000 bootstrap replicates [6] with pairwise gap deletions. All ambiguous positions were removed for each sequence pair. Branches corresponding to partitions reproduced in less than 50% bootstrap replicates are collapsed. Two *C.megacephala* OBPs (Cmeg26380, Cmeg28108) were leaved out when calculating the common cites between two sequences in multiple aligments as they may incomplete or improperly annotated sequences.

References

1. Hekmat-Scafe, D.S., et al., Genome-wide analysis of the odorant-binding protein gene family in *Drosophila melanogaster*. Genome Res, 2002. 12(9): p. 1357-69.

2. Larkin, M.A., et al., Clustal W and Clustal X version 2.0. Bioinformatics, 2007. 23(21): p. 2947-2948.

3. Tamura K., Stecher G., Peterson D., Filipski A., and Kumar S. (2013). MEGA6: Molecular Evolutionary Genetics Analysis version 6.0. Molecular Biology and Evolution30: 2725-2729

4. Saitou N. and Nei M. (1987). The neighbor-joining method: A new method for reconstructing phylogenetic trees. Molecular Biology and Evolution 4:406-425.

5. Nei M. and Kumar S. (2000). Molecular Evolution and Phylogenetics. Oxford University Press, New York.

6. Felsenstein J. (1985). Confidence limits on phylogenies: An approach using the bootstrap. Evolution 39:783-791.
