# Supplementary material for: The developmental transcriptome of the synanthropic fly Chrysomya megacephala and insights into olfactory proteins
Source: BMC Genomics. 2015 Jan 23;16(1):20. doi: 10.1186/s12864-014-1200-y (PMC4311427; doi:10.1186/s12864-014-1200-y)
Supplement: Additional file 16: — S16-Mutiple alignment of deduced amino acid of the Cmeg23484_c0 , Cmeg32081_c4 and Cmeg33593_c0 . [file 12864_2014_1200_MOESM16_ESM.pdf]

```
Cmeg23484_c0    MKATLAILI.ALFALVSA.EYKLRTQEDLVKARKECMEAKKVSAELIEKYKKFEFPDDEVTRCYIECIFEKFELFDAKDG      78
AID61300        MKASIAILI.ALFALVSA.EYKLRNQEDLMKARKECMEAKKVSPELIEKYKKFDFPDDEVTRCYIECIFEKFQLFDPKDG      78
BAN59723        MKATIAILL.ALFALVSA.DYKLRNQEDLMKARKECMEAKKVTAELIEKYKKFDFPDDEITRCYIECIFEKFQLFDPKDG      78
DmelOBP44a      MKNAVAILLCALLGLASASDYKLRTAEDLQSARKECAASSKVTEALIAKYKTFDYPDDDITRNYIQCIFVKFDLFDEAKG      80
Consensus       mk   ail  al  l sa  yklr  edl  arkec    kv   li kyk f  pdd  tr yi cif kf lfd   g

Cmeg23484_c0    FKNDNLVAQLGHGKEKKDEVKADVEKCADKNEQKSDSCAWAFRGFKCFISKNLPLVMESLKK                       140
AID61300        FKNENLITQLGHGKENKDEVKADVEKCADKNEQKTDSCAWAFRGFKCFISKNLPLVMESLKK                       140
BAN59723        FKNENLVAQLGAGKENKDEVKADVEKCADKNEQKSDSCTWAFRGFKCFISKNLPLVMESLKK                       140
DmelOBP44a      FKVENLVAQLGQGKEDKAALKADIEKCADKNEQKSPANEWAFRGFKCFLGKNLPLVQAAVQK                       142
Consensus       fk  nl  qlg gke k   kad ekcadkneqk     wafrgfkcf  knlplv     k


Cmeg32081_c4    ......MKFLLVLSVVILAACNIR..ADLTKEEAIAIATGCKEEAGASDDDFEAMIKHQPADTKEGKCMRACALKKFGVM      72
AID61308        ......MKFLIVSGFLILAACSIR..AELTKEEAIAIATDCKEEAGASDADFEAMVTHQPAISPEGKCMHACALKKFGVL      72
DmelOBP19d      MSHLVHLTVLLMVGILCLGATSAKPHEEINRDHAAELANECKAETGATDEDVEQLMSHDLPERHEAKCLRACVMKKLQIM      80
Consensus                l       l a             a   a  ck e ga d d e    h      e kc  ac  kk

Cmeg32081_c4    SDEGKMIKDAAIELSKTLIKDD.DKKALVAGVIEACEGLEVSEDHCEAAEEYGHCLKQEFESKGISSAEDLI             143
AID61308        SDEGKLLKDAALELSESLIKNE.EKKALIAEIVETCDQLEVSDDHCEAAEEYGQCWRNEFEAKGISPDEDLV             143
DmelOBP19d      DESGKLNKEHAIELVKVMSKHDAEKEDAPAEVVAKCEAIETPEDHCDAAFAYEECIYEQMKEHGLELEEH..             150
Consensus          gk  k  a el     k    k    a     c   e   dhc aa  y  c        g    e


Cmeg33593       .MKVFLLLILAVAALA.......HHEHGHHDHQDGHDYVVKHHADLVKYREDCGNKLKISPELMEKYKNWEYPDDETTHC      72
AID61305        MKVFFVILVLTVAALADHHEGHDHHDHDHHVHHESHDYVVKHHDDLVKFRDECSTKLKISPELMEKYKSWEYPDDEATHC      80
DmelOBP99b      .MKVLIVLLLGLAFVL........ADHPHHH....HDYVVKTHEDLTNYRTQCVEKVHASEELVEKYKKWQYPDDAVTHC      67
Consensus              l l  a             h hh     hdyvvk h dl   r  c  k   s el ekyk w ypdd  thc

Cmeg33593       YMKCIFEHFGFFDEHKGFDVHKIHHQLVGEHGAVDHNDETHAKIEKCADKNTQGSDACTWAYRGGMCFIQSNIQLVKDSV     152
AID61305        YMKCIFEHFGFFDEHKGFDVHKIHHQLVGEHVTVDHNDETHHKIEHCADKNTQGSDACTWAYRGGMCFIRSNLQLVKGSV     160
DmelOBP99b      YLECIFQKFGFYDTDHGFDVHKIHIQLAGPGVEVHESDEVHQKIAHCAETHSKEGDSCSKAYHAGMCFMNSNLQLVQHSV     147
Consensus       y  cif  fgf d   gfdvhkih ql g    v   de h ki  ca       d c  ay  gmcf  sn qlv  sv

Cmeg33593       HK                                                                                  154
AID61305        HK                                                                                  162
DmelOBP99b      KV                                                                                  149
Consensus
```
